# Supplementary material for: Exploring Nurse Dyads’ experiences of scope of practice in nursing homes: A qualitative descriptive study from the FLORENCE project
Source: PLoS One. 2025 Oct 10;20(10):e0334124. doi: 10.1371/journal.pone.0334124 (PMC12513666; doi:10.1371/journal.pone.0334124)
Supplement: S2 Table — (DOCX) [file pone.0334124.s002.docx]

| **Theme 1: The Context of Practice** | | |
| --- | --- | --- |
| *Duties vs Chores* | | |
| **RN** | **Dyad 1**  (S2:1) | **Non-RN** |
| ‘*We have to make sure that the quality and reporting systems are updated… the decision-making office need functional assessment… all those standardised routines, most of them fall on the registered nurses…* ’ |  | ‘*Then it is you who has all the residents, the whole kitchen, all the dishes, and laundry... the whole day… you get tired, the days are hectic. That's what makes you tired over time.* ’ |

| *Dwindling resources* | | |
| --- | --- | --- |
| **RN** | **Dyad 3**  (S2:2) | **Non-RN** |
| ‘*The nursing home is characterized by periods with few permanent nurses, which means that the tasks my colleagues and I perform, or my nurse colleagues and I perform, are more administrative. There is a nursing shortage, and we can´t maintain it, we don´t get enough people to do the job...*’ |  | ‘*Something goes back when it comes to standards and such. What is good enough. Because we often hear that it's just 'it's good enough.' We are quite tired of hearing that, you could say. That 'good enough.' I think it's idiotic to say that. It was better before. Yes, we had more resources before.* ’ |

| *Environmental challenges* | | |
| --- | --- | --- |
| **RN** | **Dyad 6**  (S2:3) | **Non-RN** |
| *‘I feel that I constantly have to follow up all the time, because some messages are missed. That is, I could have said, this resident ‘need this and that follow-up, can you keep an eye on it?' I am off Saturday-Sunday, but on Monday nothing has been done.... Have they assessed it...So, there are constant interruptions for things that wouldn't have happened or been picked up that creates more work for me…*’ |  | ‘*I get so tired… I´m kind of disappointed… There is the expectation that we have to work a bit fast... Finish as quickly as possible… upset... That they [i.e. management] expect a bit more from us… It's actually very stressful… That is the expectation, and that is the culture. And it speaks for itself. We don't have time to observe and spend time [with the residents] when it's stressful. The heart is pounding; my pulse is probably at 150...*’ |

| **Theme 2: The Nurses´ Professional Stance** |
| --- |
| *Professional Conduct* |

| **RN** | **Dyad 3**  (S2:4) | **Non-RN** |
| --- | --- | --- |
| *‘Well, it's like... It's about building responsibility. It's like I feel a greater responsibility than I perhaps did before… I feel the nursing home is dependent on me…’* |  | *‘I am proud of my job and proud of my colleagues and what we achieve. I really am. I am proud of what we achieve…’* |
| *Professional values vs. work morale* | | |
| **RN** | **Dyad 6**  (S2:5) | **Non-RN** |
| *‘I don't always feel like a good nurse when I finish my job. But I do the best I can… I think it most likely becomes very exhausting in the long run. Having to double-check people who have gone to school and are supposed to have that competence…’* |  | *‘That there's a culture... Put on the diaper, wash the lower body, done. No discussion. I have to defend the residents, that I always have to be a voice for them [the residents]….I miss a bit more professional depth. Skilled professionals. Good skilled professionals. That we can discuss, maybe we can learn a bit from each other… We need to use things positively instead of thinking negatively. We are different, with different opinions. No, you don't need to think that way. You can learn from each other… We have very skilled people…’* |
| **Theme 3: The Scope of Practice in Relation to Residents' General Care** | | |
| *The whys (of general scope of practice)* | | |
| **RN** | **Dyad 4**  (S2:6) | **Non-RN** |
| *‘Yes, we do have responsibilities as nursing tasks. If I am responsible for the whole house... If I work during the day, I take reports and read journals, and then there are some tasks specific to nursing that need to be performed during the shift. If I am on the ward, it is a bit different. Distributing medication and then care… It's very clear to me [the RNs role and function], but it's a bit difficult to express the meaning. I'm not very clear on what my role is and what I do. So, I do it every day. I do a good job here as a nurse. But it's a bit difficult to explain, right. The meaning...’* |  | *‘So, I often go with them [i.e. RNs] to meetings to fetch... If I have time, that is. To fetch medications or something like that. So, we cooperate in that way. We help each other. I don't know what their tasks are, but I assume they have a lot to do… We are with them a lot. We give a lot of ourselves and care a lot about them. We do that. We do everything we can, in a way... I feel that. As much as we can. So,... We take good care of them. We are very committed.’* |
| *The what’s (of general scope of practice)* | | |
| **RN** | **Dyad 2**  (S2:7) | **Non-RN** |
| *‘When you are responsible for everything there is no clearly marked boundary for what your role is and what is strictly speaking a bit outside of it, and then it turn out that you do a lot of things that are not a nursing task, but there is nobody else to do it, and then you twist and turn it to mange to define it within your general practice, often resulting ending up doing everything’* |  | *‘Who takes responsibility for the kitchen? For filing the cupboards with clothes for the residents? And then the utility room, which is separate, with garbage and laundry… clean the utility room, there are many clothes that needs to be washed, folded and put away in the rooms… who take care of that?’* |
|  | | |

| **Theme 4: Scope of Practice in Relation to the Residents’ Fundamentals of Care** | | | | |
| --- | --- | --- | --- | --- |
| *The what’s (of scope of practice related to FoC)* | | | | |
| **RN** | **Dyad 5**  (S2:8) | | | **Non-RN** |
| *‘It is really about dealing primarily with the fundamentals, then. Not so much with how you address other needs outside of the physical, right… So, it's just about the fundamental needs. Elimination, sleep, food, nutrition... Not so much about the... The other type… In a way... I'm not saying that the other needs are not important…*’ |  | | | *‘My role here is to ensure that the residents' needs are met... Mostly, or all of their needs. That includes personal care, feeding, activities, the social aspect, communication, talking... And with relatives, when they come…’* |
| **Theme 5: Notions About Each Other’s Scope of Practice** | | | | |
| *The ‘other´s’ why´s* | | | | |
| **RN** | **Dyad 7**  (S2:9) | | | **Non-RN** |
| *‘I feel that in my role, it’s not just about the residents, but I also have a role with my colleagues, in terms of guiding them and being able to train them in these things. I think that knowledge is power, and it should be shared… When I am in the ward, I have the same function as them… [i.e. Non-RNs] ’* |  | | | *‘Nurses have more knowledge than healthcare workers. That’s what I think. I believe that maybe there is not a very big difference, perhaps just a small difference between nurses and healthcare workers, because even nurses here also have roles related to residents’ nutrition and activities for residents, as we collaborate with them, right?’* |
| *The ‘other´s’ what´s* | | | | |
| **RN** | **Dyad 8**  (S2:10) | | | **Non-RN** |
| *‘Most is done by healthcare workers, so they inform me so that I can come and administer medication if necessary. Yes, but they are the ones who mostly talk to and calm the residents, and ensure that the psychosocial situation is managed… healthcare workers are the ones who mostly talk to the residents and build that relationship with them…’* |  | | | *‘Nursing practice, yes... We also have very good cooperation with them [i.e. RNs], and it is about helping each other, of course. I think it's fine when we call, for example, if a resident is unwell, if a resident needs some medication or extra painkillers like strong medications. We can't give the strong medications, they come, or they bring the medications. That's what we have. Good cooperation’* |
| *Absence vs. presence* | | | | |
| **RN** | | **Dyad 1**  (S2:11) | **Non-RN** | |
| *‘I might not be the one who are the most present in the department and in the care, that you are not present in all the care situations that some might feel you should be’* | |  | *The RNs are with the residents too, but I feel that it is we healthcare workers who are more with the resident throughout the day. It is we who observe more. We are more with the residents then... The RNs come, maybe only if we need help. Or if there is something, then we call, and they might come for a minute or two and then they leave again.’* | |
|  | |  |  | |

_______________________

**Abbreviations:**

FoC = Fundamentals of Care

Non-RN = non-Registered Nurse

RN = Registered Nurse
