# Supplementary material for: Exploring Nurse Dyads’ experiences of scope of practice in nursing homes: A qualitative descriptive study from the FLORENCE project
Source: PLoS One. 2025 Oct 10;20(10):e0334124. doi: 10.1371/journal.pone.0334124 (PMC12513666; doi:10.1371/journal.pone.0334124)
Supplement: Attachment — Submitted filename: PONE-D-25-27718 (1).docx [file pone.0334124.s003.docx]

PLOS ONE

**Exploring Nurse Dyads’ Experiences of Scope of Practice in Nursing Homes: A Qualitative Descriptive Study from the FLORENCE Project**

--Manuscript Draft--

| **Manuscript Number:** | PONE-D-25-27718 |
| --- | --- |
| **Article Type:** | Research Article |
| **Full Title:** | Exploring Nurse Dyads’ Experiences of Scope of Practice in Nursing Homes: A Qualitative Descriptive Study from the FLORENCE Project |
| **Short Title:** | Nurses Professional Scope of Practice in Nursing Homes: the FLORENCE project |
| **Corresponding Author:** | Ole Martin Nordaunet  Karlstad University: Karlstads Universitet Karlstad, SWEDEN |
| **Keywords:** | Dyads, scope of practice, fundamentals of care, nursing homes, registered nurses, non-registered nurses, qualitative research, framework method |
| **Abstract:** | Background  A nurse’s scope of practice encompasses the full spectrum of roles, functions, responsibilities, and decision-making capacity. However, the scope of practice  —particularly in relation to the fundamentals of care— remains underexplored from the perspective of registered- and non-registered nurses working in nursing homes. This study aimed to explore nurses’ experiences of their mutual general scope of practice, the differences in their individual scopes, and their respective scope of practice in relation to the fundamentals of care.  Methods  This qualitative study included eight dyads of registered nurses (RNs) and non- registered nurses (non-RNs) (n = 16) working in four nursing homes in south-east Norway. Non-dyadic qualitative data were collected through focused interviews and analysed using the framework method for dyadic analysis. The study was reported in accordance with the Standards for Reporting Qualitative Research (SRQR).  Results  The context of practice emerged as a critical factor modulating the professional practice environment, as mutually described by the nurses. Under pressure, nurses often adopted strategies that simplified their scope of practice, albeit at the expense of their professional stance. While RNs’ scope of practice primarily involved indirect care, non-RNs were more engaged in direct care—especially in relation to the fundamentals of care. Non-RNs described addressing residents’ physical, relational, and psychosocial needs, whereas RNs, although acknowledging the importance of holistic care, focused mainly on physical needs. RNs were frequently reliant on delegating tasks to NRNs, a practice that introduced both concerns and challenges.  Conclusion  The dyadic perspective provided a comprehensive understanding of the scope of practice within nursing homes, highlighting the complexity of nursing responsibilities and activities in long-term care settings. There is a need for strategies that enhance nurses’ understanding of their distinct scopes of practice, reinforce the delivery of fundamental care, strengthen professional identity, and support effective delegation practices. |
| **Order of Authors:** | Ole Martin Nordaunet |
|  | Hanne Aagaard, PhD |
|  | Cecilia Olsson, PhD |
|  | Edith Roth Gjevjon, PhD |
|  | Gunilla Borglin, PhD |
| **Additional Information:** |  |
| **Question** | **Response** |
| **Financial Disclosure** | The author(s) received no specific funding for this work. |

| Enter a financial disclosure statement that describes the sources of funding for the work included in this submission. Review the [submission guidelines](http://journals.plos.org/plosone/s/submission-guidelines#loc-financial-disclosure-statement) for detailed requirements. View published research articles from [*PLOS ONE*](https://journals.plos.org/plosone/) for specific examples.  This statement is required for submission and **will appear in the published article** if the submission is accepted. Please make sure it is accurate. |  |
| --- | --- |
| **Funded studies**  Enter a statement with the following details:   - Initials of the authors who received each award - Grant numbers awarded to each author - The full name of each funder - URL of each funder website - Did the sponsors or funders play any role in the study design, data collection and analysis, decision to publish, or preparation of the manuscript? |  |
| Did you receive funding for this work? |  |
| **Competing Interests**  Use the instructions below to enter a competing interest statement for this submission. On behalf of all authors, disclose any [competing interests](http://journals.plos.org/plosone/s/competing-interests) that could be perceived to bias this work—acknowledging all financial support and any other relevant financial or non- financial competing interests.  This statement is required for submission and **will appear in the published article** if the submission is accepted. Please make sure it is accurate and that any funding sources listed in your Funding Information later in the submission form are also declared in your Financial Disclosure statement. | The authors have declared that no competing interests exist. |

| View published research articles from  [*PLOS ONE*](http://journals.plos.org/plosone/) for specific examples. |  |
| --- | --- |
| **NO authors have competing interests**  Enter: *The authors have declared that no competing interests exist.*  **Authors with competing interests**  Enter competing interest details beginning with this statement:  *I have read the journal's policy and the authors of this manuscript have the following competing interests: [insert competing interests here]* |  |
| * typeset |  |
| **Ethics Statement**  Enter an ethics statement for this submission. This statement is required if the study involved:   - Human participants - Human specimens or tissue - Vertebrate animals or cephalopods - Vertebrate embryos or tissues - Field research   Write "N/A" if the submission does not require an ethics statement.  General guidance is provided below. Consult the [submission guidelines](http://journals.plos.org/plosone/s/submission-guidelines#loc-guidelines-for-specific-study-types) for detailed instructions. **Make sure that all information entered here is included in the Methods section of the manuscript.** | This study was assessed and given a waiver by relevant authorities in Norway (Regional Ethics Committees ID 691021/2024) and the Norwegian Agency for Shared Services in Education and Research (ID 524471/2024) as well as in Sweden (Research Ethics committee, Karlstad University Sweden (ID C2024/55) and the Swedish Ethical Review Authority (ID 2024-000586-01).  Participants were recruited between 28th of February to 4th of June 2024. All participants provided informed consents and were informed verbally and in writing before signing their consent. |

| **Format for specific study types**  **Human Subject Research (involving human participants and/or tissue)**   - Give the name of the institutional review board or ethics committee that approved the study - Include the approval number and/or a statement indicating approval of this research - Indicate the form of consent obtained (written/oral) or the reason that consent was not obtained (e.g. the data were analyzed anonymously)   **Animal Research (involving vertebrate animals, embryos or tissues)**   - Provide the name of the Institutional Animal Care and Use Committee (IACUC) or other relevant ethics board that reviewed the study protocol, and indicate whether they approved this research or granted a formal waiver of ethical approval - Include an approval number if one was obtained - If the study involved *non-human primates*, add *additional details* about animal welfare and steps taken to ameliorate suffering - If anesthesia, euthanasia, or any kind of animal sacrifice is part of the study, include briefly which substances and/or methods were applied   **Field Research**  Include the following details if this study involves the collection of plant, animal, or other materials from a natural setting:   - Field permit number - Name of the institution or relevant body that granted permission |  |
| --- | --- |
|  |  |
| **Data Availability**  Authors are required to make all data underlying the findings described fully available, without restriction, and from the time of publication. PLOS allows rare exceptions to address legal and ethical concerns. See the [PLOS Data Policy](http://journals.plos.org/plosone/s/data-availability) and [FAQ](http://journals.plos.org/plosone/s/data-availability#loc-faqs-for-data-policy) for detailed information. | No - some restrictions will apply |

| A Data Availability Statement describing where the data can be found is required at submission. Your answers to this question constitute the Data Availability Statement and **will be published in the article**, if accepted. |  |
| --- | --- |
| **Important:** Stating ‘data available on request from the author’ is not sufficient. If your data are only available upon request, select ‘No’ for the first question and explain your exceptional situation in the text box. |  |
| Do the authors confirm that all data underlying the findings described in their manuscript are fully available without restriction? |  |
| **Describe where the data may be found in full sentences. If you are copying our sample text, replace any instances of XXX with the appropriate details.**   - If the data are **held or will be held in a public repository**, include URLs, accession numbers or DOIs. If this information will only be available after acceptance, indicate this by ticking the box below. For example: *All XXX files are available from the XXX database (accession number(s) XXX, XXX.*). - If the data are all contained **within the manuscript and/or Supporting Information files**, enter the following: *All relevant data are within the manuscript and its Supporting Information files.* - If neither of these applies but you are able to provide **details of access elsewhere**, with or without limitations, please do so. For example:   *Data cannot be shared publicly because of [XXX]. Data are available from the XXX Institutional Data Access / Ethics Committee (contact via XXX) for researchers who meet the criteria for access to confidential data.*  *The data underlying the results presented in the study are available from (include the name of the third party* | Data cannot be shared publicly because of confidential and protected data as approval given by the research authorities. |

| *and contact information or URL).*   - This text is appropriate if the data are owned by a third party and authors do not have permission to share the data.   * typeset |  |
| --- | --- |
| Additional data availability information: |  |

Main document with tables	[Click here to access/download;Manuscript;Main document](https://www2.cloud.editorialmanager.com/pone/download.aspx?id=40557738&guid=aa51fd76-3af7-4e79-aff3-1a19bb520d09&scheme=1)[<https://www2.cloud.editorialmanager.com/pone/download.aspx?id=40557738&guid=aa51fd76-3af7-4e79-aff3-1a19bb520d09&scheme=1> 23.05.25.docx](https://www2.cloud.editorialmanager.com/pone/download.aspx?id=40557738&guid=aa51fd76-3af7-4e79-aff3-1a19bb520d09&scheme=1)

1 Exploring Nurse Dyads’ Experiences of Scope of Practice in Nursing Homes: A Qualitative Descriptive
2 Study from the FLORENCE Project

3

4	Nurses Professional Scope of Practice in Nursing Homes: the FLORENCE project

5

6

7 Nordaunet O.M., RN, MSc, PhD-student,^1,2^ Aagaard H., Professor,^1^ Olsson C., Associate Professor^,1,2^ Gjevjon E. R.,
8 Professor of Nursing,^1,3^ Borglin G., Professor of Nursing^1^

9

10 ^1^Lovisenberg Diaconal University College, Department of Bachelor Education (Nursing). Lovisenberggata 15B, NO-
11 0456 Oslo, Norway
12 2 Karlstad University, Institute of Health Sciences, Department of Nursing. Universitetsgatan 2, 651 88 Karlstad,
13 Sweden
14 ^3^UiT The Arctic University of Norway, Department of Bachelor Education, Havnegata 5, 9404 Harstad.

15

16

17	Wordcount: 6225

18

19 Ole Martin Nordaunet: <https://orcid.org/0000-0002-6061-0033>
20 Edith Roth Gjevjon: <https://orcid.org/0000-0002-9656-522X>
21 Cecilia Olsson: <https://orcid.org/0000-0002-0944-5650>
22 Hanne Aagaard: <https://orcid.org/0000-0002-7810-9298>
23 Gunilla Borglin: <https://orcid.org/0000-0002-7934-6949>

24

25 Corresponding author:
26 Ole Martin Nordaunet
27 Karlstad University, Institute of Health Sciences, Department of Nursing
28 Universitetsgatan 2, 651 88 Karlstad, Sweden
29 E-mail: [Ole.martin.nordaunet@ldh.no](mailto:Ole.martin.nordaunet@ldh.no)
30 Phone: + 47 997 313 70

31

32

33

34

35

36

37 ABSTRACT
38 Background
39 A nurse’s scope of practice encompasses the full spectrum of roles, functions, responsibilities, and decision-making
40 capacity. However, the scope of practice —particularly in relation to the fundamentals of care— remains
41 underexplored from the perspective of registered- and non-registered nurses working in nursing homes. This study
42 aimed to explore nurses’ experiences of their mutual general scope of practice, the differences in their individual
43 scopes, and their respective scope of practice in relation to the fundamentals of care.
44 Methods
45 This qualitative study included eight dyads of registered nurses (RNs) and non-registered nurses (non-RNs) (n =
46 16) working in four nursing homes in south-east Norway. Non-dyadic qualitative data were collected through
47 focused interviews and analysed using the framework method for dyadic analysis. The study was reported in
48 accordance with the Standards for Reporting Qualitative Research (SRQR).
49 Results
50 The context of practice emerged as a critical factor modulating the professional practice environment, as mutually
51 described by the nurses. Under pressure, nurses often adopted strategies that simplified their scope of practice,
52 albeit at the expense of their professional stance. While RNs’ scope of practice primarily involved indirect care,
53 non-RNs were more engaged in direct care—especially in relation to the fundamentals of care. Non-RNs described
54 addressing residents’ physical, relational, and psychosocial needs, whereas RNs, although acknowledging the
55 importance of holistic care, focused mainly on physical needs. RNs were frequently reliant on delegating tasks to
56 NRNs, a practice that introduced both concerns and challenges.
57 Conclusion
58 The dyadic perspective provided a comprehensive understanding of the scope of practice within nursing homes,
59 highlighting the complexity of nursing responsibilities and activities in long-term care settings. There is a need for
60 strategies that enhance nurses’ understanding of their distinct scopes of practice, reinforce the delivery of
61 fundamental care, strengthen professional identity, and support effective delegation practices.
62 Keywords: Dyads, scope of practice, fundamentals of care, nursing homes, registered nurses, non-registered
63 nurses, qualitative research, framework method

64

65

66 Introduction
67 In recent years, the concept of nurses’ scope of practice (SoP) has gained prominence on the research agenda as
68 a means to better delineate and define nursing roles. For the purposes of this study, the term "nurses" refers to
69 both registered nurses and non-registered nurses. More recently, SoP has been defined as "the full spectrum of
70 roles, functions, responsibilities, activities, and decision-making capacity which nurses are educated, competent,
71 and authorised to perform" [1] (p. 7). This definition underscores that the SoP should reflect the full breadth of
72 nursing capabilities, with particular emphasis on the role, function, and decision-making unique to each type of
73 nurse and their qualifications [2]. SoP can thus be understood as grounded in the individual nurse’s professional
74 stance, reflective capacity, and practical and problem-solving skills [3], forming the theoretical and practical
75 foundation for professional nursing.

76

77 Despite increasing calls to optimize the use of nursing resources, research continues to show that nurses' SoP
78 remains underutilized or poorly aligned with their full potential [4, 5]. This can be viewed within the broader
79 context of systemic changes in healthcare delivery—particularly the shift wherein bachelor-educated Registered
80 Nurses (RNs) have become less visible at the bedside, while less formally trained personnel, such as non-registered
81 nurses (Non-RNs), are increasingly engaged in tasks traditionally performed by RNs. This shift has reoriented the
82 RN’s role toward supervision, coordination, and administrative oversight [6]. Such changes are particularly relevant
83 for what is arguably the most frequently provided aspect of nursing care: the fundamentals of care (FoC) [7, 8].

84

85 SoP in relation to the FoC encompasses the nurse’s responsibility to address the complex and dynamic interaction
86 of a patient’s physical, psychosocial, and relational needs. This approach places the nurse’s commitment to care
87 at the centre, while framing the SoP within the care context [9]. A clear understanding of SoP in this regard is
88 critical, especially as multiple nursing roles—including RNs and non-RNs—are involved in delivering fundamental
89 care. Without such clarity, role confusion may arise, professional identity can be weakened, and the risk of
90 practicing outside one’s authorised scope increases [5, 10, 11]. This complexity is further exacerbated by the
91 ongoing global shortage of RNs [12], prompting the development of new care models that involve task-shifting
92 [13]. Such models often expand non-RNs' responsibilities while assigning RNs broader oversight over patient
93 safety, coordination, and quality assurance [2, 14]. The implications of these shifts are particularly significant in
94 facility-based long-term care settings, where skill mix is a recognised patient safety factor [15]. Additional factors
95 such as nurse-to-patient ratios and time spent per resident [16] further modulate care outcomes. Although
96 Norway has a well-developed long-term care infrastructure, characterised by a high presence of non-RNs at the
97 bedside [17], little research has examined how this affects patient safety and care quality—especially in relation
98 to FoC, despite its clinical significance [18, 19].

99

100 Given that RNs and non-RNs are the primary providers of fundamental care, these two professions collaborate
101 closely, each contributing within their respective SoP. While the professional relationship between them is
102 outlined in regulatory guidelines [6, 20], non-RNs have often been described as the “operational arm” of RNs [21].
103 In this dynamic, the RN’s role encompasses leadership, coordination, assessment, care planning, decision-making,
104 delegation, and supervision [1, 22, 23], making them accountable for the quality of care provided. However, the
105 RN’s SoP extends beyond administrative responsibilities to include the direct delivery of FoC—historically central
106 to nursing and still vital for ensuring patient safety and care quality [24, 25]. Our preliminary review of the
107 literature revealed a notable gap: while the scope of practice among specialist RNs has been frequently studied,
108 far less attention has been given to the everyday roles and responsibilities of generalist RNs—particularly in the
109 context of nursing homes. Similarly, few studies have explored how the SoP is enacted in relation to the FoC in
110 long-term care environments [18, 26].

111

112 Aim
113 This study aimed to explore the experiences of RNs and non-RNs regarding their mutual general scope of practice,
114 the differences in their individual scope of practice, and their respective scopes in relation to the fundamentals of
115 care in the context of nursing homes

116

117 Methods
118 A qualitative descriptive design [27] was chosen as a pragmatic approach to gather rich, contextualised data. Data
119 were collected through focused interviews [28], and analysed using the framework method, inspired by Collaço et
120 al. [29]. This method enabled exploration of the variations in nurses’ experiences based on their professional
121 responsibilities and activities —i.e., their scope of practice—while also identifying shared concerns, similarities,
122 and incongruences both within and across professions. The study is reported in line with the Standards for
123 Reporting Qualitative Research (SRQR) [30].

124

125 *Recruitment and Participants*
126 Participants were registered nurses (RNs) and non-registered nurses (Non-RNs)—including healthcare workers
127 and healthcare assistants—employed at four nursing homes in southeast Norway. All participants had previously
128 consented to participate in a broader two-strand research project. The first strand involved direct structured
129 observations [31], while the current strand employed a qualitative descriptive design with focused interviews. Out
130 of 58 eligible participants (24 RNs and 34 Non-RNs), 21 RNs and 10 non-RNs were purposefully recruited [32] and
131 invited to participate. Reasons for non-participation included time constraints, language barriers, and one
132 withdrawal due to personal reasons. From the recruited participants, eight RNs and eight non-RNs were selected
133 to form eight dyads, based on having worked together during the structured observation phase of the first project
134 strand. For more detailed information about the studies contextual framing please see Figure 1 in Nordaunet et
135 al., 2025 [31].

136

137 *Data Collection*
138 Non-dyadic data were collected through individual focused interviews with each member of the eight dyads,
139 ensuring separation during data collection [29]. Interviews were conducted at a mutually agreed time and place,
140 audio-recorded, and demographic/background information was also collected. The interviews centred around two
141 open-ended questions:

142

143 1.	IQ1: "Can you describe your nursing practice in general and in relation to the fundamentals of care?"
144 2.	IQ2: "Can you describe your individual and shared scope of nursing practice?"

145

146 Probing questions such as “Can you give me an example?”, “Can you explain in more detail?”, and “How did you
147 think, feel, or reflect?” were used to encourage elaboration. All interviews were transcribed verbatim using GDPR-
148 compliant transcription software [33] and secure data storage [34]. Data collection occurred between March and
149 June 2024. RN interviews ranged from 37 to 66 minutes (mean: 54 minutes), and NRN interviews ranged from 39
150 to 71 minutes (mean: 50 minutes). The average interview length across all interviews (n = 16) was 52 minutes.

151

152 *Data Analysis*
153 The transcribed text was analysed using a dyadic approach informed by the Framework Method, with dyads
154 serving as the primary unit of analysis [29]. The method followed eight iterative stages. After transcription (Stage
155 1) and familiarisation with the data (Stage 2), content relevant to the interview foci was extracted (Stage 3),
156 entered into a structured dyad template, and coded. To assess interrater reliability, one dyad was randomly
157 selected, and each member of the research team independently extracted quotes addressing interview questions
158 1 and 2 from the first three pages of the dyad interviews (RN and Non-RN). This process resulted in 93.3%
159 agreement across extractions. In Stage 4, codes were organised into a chart to inform the subsequent analysis
160 (Stage 5), which in turn served as the basis for developing an analytical framework (Stage 6). This framework was
161 iteratively refined (Stage 7) and used to guide the interpretation of the data (Stage 8), drawing on insights
162 accumulated throughout all prior stages (see Table 1).

163

164 Stages 1–4 were conducted independently for each professional group. From Stage 5 onward, codes were sorted
165 and compared across professions, allowing for analysis of similarities and differences within and between groups.
166 Codes and themes were refined iteratively as familiarity with the data deepened. The research team collaborated
167 closely throughout the process, ensuring each stage was completed before moving on to the next.

168

169	Table 1. Overview of the process for the framework analysis

170

General theme III: SoP in Relation to Residents´ General Care

DYAD 1	Registered nurse	Non-registered nurse

*Interview Question	Transcript quote 19	Coding	Transcript quote 1	Coding	Characteristics*

1	“I feel that my role is to have a bit of overview… one become very focused on the medical part… and to bring the medical aspect into the care part.”

Keeping the overview Focused on medicinal care

Bringing medical aspect into care

“I am here to help those who are here, those who can’t manage at home themselves. Those who don’t have any relatives to help them. Their daily needs. To dress them and feed them and make sure they are well…. I would say we are caregivers. We are here to help the patients with their needs, both daily, spiritual, social and physical needs.”

Helping those who can´t manage

Daily needs

Hygiene and dressing Eating and drinking Holistic care

*The why´s in general scope of practice*

*The what’s and the*

*how’s in general scope of practice*

General theme IV: SoP in Relation to the Residents´ FoC

DYAD 4	Registered nurse	Non-registered nurse

*Interview Question	Transcript quote 5	Coding	Transcript quote 3	Code	Characteristics*

1 “I arrange conversations with the relatives and then I do it twice a year. Other tasks, for example as a nursing task... Ensuring that the residents are well here. In terms of hygiene, like medications… We create measures so they can go for a walk once, two or three times a week.”

Involve and inform relatives

Hygiene and dressing Medication management Mobility

Create tasks in care plan

“I often say, 'good morning' and 'did you sleep well?'. Many of them recognize me, I would say. It's very nice. I also sit a bit at the bedside. Someone got a cup of coffee at the bedside. A bit of contact before we start the care… I make sure to wash well… Make sure the water is warm… Make sure they sit on the toilet… There's care and medication and... Yes, if you think like that, yes. Care and medication.”

Introducing, friendly Sitting down bedside Eating and drinking Hygiene and dressing Medication management

*The what’s of the fundamentals of care*

General theme V: Notions About Each-other’s SoP

DYAD 8	Registered nurse	Non-registered nurse

*Interview Question	Transcript quote 13	Coding	Transcript quote 7	Coding	Characteristics*

2 “The difference between RN and HCW is actually nothing... The only thing is that nurses are mostly focused on medication management. Otherwise, the rest is the same. No difference, except you have more oversight over medications…”

171

Little difference between RN and NRN RN have more competence in relation to medication management

“Nursing practice, yes... We also have very good cooperation with them, and it is about helping each other, of course. I think it's fine when we call if a patient is unwell, if a patient needs some medication or extra painkillers like strong medications. If we take the medications, but we can't give the strong medications, they come, or they bring the medications. That's what we have. Good cooperation.”

Collaborating with RNs Call the RN is patient is unwell

RNs bring medicines that NRN can´t administer

*The “other” why´s*

*The “other” what’s and the how’s*

*Absences vs. presence*

172

173

174

175

176

Abbreviations:

FoC = Fundamentals of Care RN = Registered Nurse

NRN = Non-registered nurse

177

178

179

180

181

182

183

184

185

186

187

188

189

190

191

192

193

194

195

196

197

198

199

200

201

*Ethical Considerations*

The study received ethical approval from relevant authorities in both Norway and Sweden:

- Regional Committees for Medical and Health Research Ethics, Norway (ID: 691021/2024)
- Norwegian Agency for Shared Services in Education and Research (ID: 524471/2024)
- Research Ethics Committee, Karlstad University, Sweden (ID: C2024/55)
- Swedish Ethical Review Authority (ID: 2024-000586-01)

Participants were recruited between 28^th^ of February to 4^th^ of June 2024. All participants provided informed consents and were informed verbally and in writing before signing their consent.

*Rigour and Reflexivity*

All authors are registered nurses. Four hold academic positions and have extensive experience in clinical and research settings. The first author is a PhD student with no prior detailed experience in the nursing home context; the same applies to two other team members. The remaining two authors possess extensive experience in long- term care and geriatric nursing. This diverse composition contributed to critical reflection throughout the research process. Regular discussions and reflexive practices ensured that interpretation remained grounded in the participants’ narratives, minimising the impact of preconceptions.

Results

Eight dyads participated in this study. The RNs consisted of five females and three males, with a mean age of 37.7 years (range: 28–58, SD = 11). The eight participating non-RNs were all female, with a mean age of 46.7 years (range: 32–61, SD = 10.2). The overall mean age of the informants was 42.2 years (Table 2).

Table 2. Informant characteristics

| Age Sex Working Further education Dyads (years) experience  (years) | | | | |
| --- | --- | --- | --- | --- |
| Dyad I: RN | 58 | ♀ | 11 | N/A |
| Dyad I: NRN | 37 | ♀ | 4 | N/A |
| Dyad II: RN | 34 | ♀ | 7 | N/A |
| Dyad II: NRN | 32 | ♀ | 11 | Dementia and person-centred care |
| Dyad III: RN | 41 | ♂ | 6 | Administration and leadership |
| Dyad III: NRN | 55 | ♀ | 3 | N/A |

202

| Dyad IV: RN | 30 | ♂ | 6 | Mental health |
| --- | --- | --- | --- | --- |
| Dyad IV: NRN | 61 | ♀ | 17 | Mental health |
| Dyad V: RN | 34 | ♀ | 8 | N/A |
| Dyad V: NRN | 57 | ♀ | 10 | N/A |
| Dyad VI: RN | 34 | ♂ | 1 | N/A |
| Dyad VI: NRN | 41 | ♀ | 2 | N/A |
| Dyad VII: RN | 43 | ♀ | 14 | Advanced Practice Nurse |
| Dyad VII: NRN | 44 | ♀ | 9 | N/A |
| Dyad VIII: RN | 28 | ♀ | 4 | N/A |
| Dyad VIII: NRN | 46 | ♀ | 3 | Dementia care and geriatric psychiatry |

203

204

205

206

207

208

209

210

211

212

213

214

215

216

217

218

219

220

221

222

223

224

225

226

227

228

Abbreviations:

RN = Registered nurse

NRN = non-registered nurse N/A = Not applicable

The framework method for analysis of dyadic data of the transcribed texts resulted in five general mainly congruent themes reflecting the nurses' experience of their individual and mutual general scope of practice, as well as their scope of practice related to the fundamentals of care among older people at nursing homes. Additionally, one general theme was identified, mirroring RNs’ experiences of challenges related to 'the others'.

*Theme 1: The Context of Practice*

The first mutual theme, “The context of practice,” reflected variations in nurses’ experiences of their general scope of practice, while also revealing underlying systemic challenges. This theme comprised three key characteristics: duties versus chores, dwindling resources, and environmental challenges. Each of these were interpreted as modulating the nurses' scope of practice

The distinction between duties vs chores highlighted how RNs and non-RNs experienced their responsibilities differently. RNs reported feeling trapped in a complex organisational system dominated by mandatory administrative obligations. These included tasks related to the nursing home's internal quality systems, as well as broader municipal and governmental care indicators. They described their responsibilities as representing a “fork in the road”: on one side was their professional duty to ensure high-quality care for residents; on the other, the demands of the system consumed valuable time, creating a sense of frustration and detachment from direct patient care. These obligations were experienced as both bureaucratic and exasperating. In contrast, non-RNs referred to their responsibilities as daily “chores.” Although their accounts echoed concerns about time being diverted from resident care, they focused more on routine operational duties such as maintaining cleanliness, supporting mealtimes, ensuring a well-functioning ward environment, and completing required documentation.

229 This included updating quality indicators and conducting mandatory functional assessments. Despite differing
230 roles and language, both RNs and NRNs conveyed a shared perception that systemic demands significantly limited
231 their ability to spend meaningful time with residents (Box 1).

232

Dyad 1

RN	NRN

233

234

‘*We have to make sure that the quality and reporting systems are updated… the decision-making office need functional assessment… all those standardised routines, most of them fall on the registered nurses…* ’

Box 1. Duties vs Chores

‘*Then it is you who has all the residents, the whole kitchen, all the dishes, and laundry... the whole day… you get tired, the days are hectic. That's what makes you tired over time.* ’

235 Both RNs and non-RNs reported that dwindling resources—particularly related to staffing levels and skill mix—
236 negatively affected their scope of practice. RNs primarily expressed concern about the shortage of qualified nurses
237 and the challenges of working in understaffed units. One RN described the situation starkly: “If everyone breathes
238 at the end of the shift, you are satisfied” (Dyad 1), highlighting the high-pressure environment and reduced
239 capacity to provide comprehensive care. Non-RNs also addressed issues related to staffing, but their concerns
240 encompassed a broader range of factors. In addition to echoing the RNs’ concerns about inadequate staffing and
241 the impact on care quality, non-RNs described the implications of limited budgets, noting how financial constraints
242 restricted staffing flexibility and compromised the overall safety and well-being of residents. Their accounts
243 illustrated a wider perspective on systemic limitations and resource allocation (Box 2).

244

Dyad 3

RN	NRN

245

246

‘*The nursing home is characterized by periods with few permanent nurses, which means that the tasks my colleagues and I perform, or my nurse colleagues and I perform, are more administrative. There is a nursing shortage, and we can´t maintain it, we don´t get enough people to do the job...*’

Box 2. Dwindling resources

‘*Something goes back when it comes to standards and such. What is good enough. Because we often hear that it's just 'it's good enough.' We are quite tired of hearing that, you could say. That 'good enough.' I think it's idiotic to say that. It was beter before. Yes, we had more resources before.* ’

247 The theme *“Context of practice”* also highlighted environmental challenges faced by both RNs and non-RNs. While
248 the concerns were similar, non-RNs expressed a broader range of issues. RNs primarily described their scope of
249 practice as hectic and stressful, marked by time pressure and a constant need to move between tasks. In contrast,
250 non-RNs not only shared these concerns but also reported feeling the additional burden of an overwhelming
251 workload. Their experiences reflected a sense of exhaustion, with many describing the feeling of carrying the
252 entire workload on their own, which contributed to a heightened sense of pressure and fatigue (Box 3).

253

Dyad 6

RN	NRN

254

255

256

*‘I feel that I constantly have to follow up all the time, because some messages are missed. That is, I could have said, this resident ‘need this and that follow-up, can you keep an eye on it?' I am off Saturday-Sunday, but on Monday nothing has been done.... Have they assessed it...So, there are constant interruptions for things that wouldn't have happened or been picked up that creates more work for me…*’

Box 3. Environmental challenges

*Theme 2: the Nurses’ professional stance*

‘*I get so tired… I´m kind of disappointed… There is the expectation that we have to work a bit fast... Finish as quickly as possible… upset... That they [i.e. management] expect a bit more from us… It's actually very stressful… That is the expectation, and that is the culture. And it speaks for itself. We don't have time to observe and spend time [with the residents] when it's stressful. The heart is pounding; my pulse is probably at 150...*’

257 The theme "The Nurses' Professional Stance" reflected a mutual understanding in how nurses experienced their
258 general scope of practice but also highlighted incongruencies in two main characteristics: Professional
259 Conduct and Professional Values vs. Working Morale, which were interpreted as constituting the theme.

260

261 The RNs described their scope of practice in a way that reflected professional conduct characterized by taking
262 shortcuts, even though they knew how to perform tasks properly and were aware of the expected practices. The
263 reasons they provided for this behaviour included a desire to avoid burnout, lack of time to do things properly, or
264 feeling that they lacked the capacity to do so. One RN from Dyad 1 stated:

265

266 *"So, you overlook that thing because another thing is more important, in a way. And because you can't be bothered*
267 *to fight all the battles all the time. And that you're a bit lazy. I don't know."*

268

269 In contrast, the Non-RNs expressed a scope of practice where their professional conduct was in clear incongruence
270 with the RNs' conduct. The non-RNs emphasized the importance of teamwork, collaborative efforts, and the need
271 to cooperate more, regardless of professional titles. They also highlighted the importance of individual conduct,
272 which included being ethically considerate, empathetic, contributing to a positive working environment, and
273 taking responsibility. Furthermore, non-RNs saw it as part of their general scope of practice to support RNs by
274 easing tasks such as medication management, staying professional in challenging situations, and displaying pride
275 in their work (Box 4).

276

Dyad 3

RN	NRN

277

278

*‘Well, it's like... It's about building responsibility. It's like I feel a greater responsibility than I perhaps did before… I feel the nursing home is dependent on me…’*

Box 4. Professional conduct

*‘I am proud of my job and proud of my colleagues and what we achieve. I really am. I am proud of what we achieve…’*

279 The theme ‘Nurses' Professional Stance’ also encompassed experiences related to the nurses' professional values
280 vs. their working morale. This characteristic was experienced by both RNs and non-RNs in ways that were mutual
281 yet reflected some variation. RNs described a sense of never feeling that their work was good enough, often not
282 feeling like a competent nurse, experiencing guilt, and striving to do their best to get everything done. The non-
283 RNs' descriptions were similar to those of the RNs, with the main variation being in how the non-RNs viewed their
284 responsibilities. The non-RNs emphasized concerns about how their professional values were compromised and
285 expressed disappointment in the care culture, or the way care was delivered, which they perceived as ethically
286 questionable (Box 5).

287

Dyad 6

RN	NRN

288

289

*‘I don't always feel like a good nurse when I finish my job. But I do the best I can… I think it most likely becomes very exhausting in the long run. Having to double-check people who have gone to school and are supposed to have that competence…’*

Box 5. Professional values vs. work morale

*‘That there's a culture... Put on the diaper, wash the lower body, done. No discussion. I have to defend the residents, that I always have to be a voice for them [the residents]….I miss a bit more professional depth. Skilled professionals. Good skilled professionals. That we can discuss, maybe we can learn a bit from each other… We need to use things positively instead of thinking negatively. We are different, with different opinions. No, you don't need to think that way. You can learn from each other… We have very skilled people…’*

290 *Theme 3: The Scope of Practice in Relation to Residents' General Care*
291 The mutual theme, ‘The Scope of Practice in Relation to Residents’ General Care’, highlighted an incongruency
292 across two main characteristics: The Whys and The What’s.

293

294 The first characteristic, The Whys, primarily reflected the RNs' descriptions of their professional responsibilities,
295 particularly their role. RNs expressed that they were tasked with solving problems on a daily basis, managing the
296 overall daily operation, and ensuring that residents' needs were met in an adequate manner. Their experience
297 also included responsibilities for prioritizing activities and tasks, as well as transferring accountability—such as
298 delegating tasks to others. RNs noted that they often had to forward unfinished tasks to the next shift. In contrast,
299 the non-RNs' descriptions of their responsibilities centered more on assisting the RNs with minor tasks, but also
300 involved closer engagement with residents, such as being with them throughout the day, sitting down with them,
301 holding hands, and offering emotional support (Box 6).

302

Dyad 4

RN	NRN

303

304

*‘Yes, we do have responsibilities as nursing tasks. If I am responsible for the whole house... If I work during the day, I take reports and read journals, and then there are some tasks specific to nursing that need to be performed during the shift. If I am on the ward, it is a bit different. Distributing medication and then care… It's very clear to me* [*the RNs role and function*]*, but it's a bit difficult to express the meaning. I'm not very clear on what my role is and what I do. So, I do it every day. I do a good job here as a nurse. But it's a bit difficult to explain, right. The meaning...’*

Box 6. The whys *(of general scope of practice)*

*‘So, I often go with them* [*i.e. RNs*] *to meetings to fetch... If I have time, that is. To fetch medications or something like that. So, we cooperate in that way. We help each other. I don't know what their tasks are, but I assume they have a lot to do… We are with them a lot. We give a lot of ourselves and care a lot about them. We do that. We do everything we can, in a way... I feel that. As much as we can. So,... We take good care of them. We are very committed.’*

305 The second characteristic, The What's, predominantly reflected the activities and tasks, or their function, as
306 described by both RNs and non-RNs. These were seen as part of their general scope of practice within the nursing
307 home context. Both RNs and non-RNs described their primary engagement in clinical observations and
308 assessments. Additionally, the non-RNs emphasized their involvement in delivering daily care, such as addressing
309 residents' physical needs and supporting the maintenance of their functional abilities. Non-RNs also described
310 activities such as forwarding information to the RNs and updating documentation in residents' journals as part of
311 their scope of practice (Box 7).

312

Dyad 2

RN	NRN

313

314

*‘When you are responsible for everything there is no clearly marked boundary for what your role is and what is strictly speaking a bit outside of it, and then it turn out that you do a lot of things that are not a nursing task, but there is nobody else to do it, and then you twist and turn it to mange to define it within your general practice, often resulting ending up doing everything’*

Box 7. The what’s (*of general scope of practice*)

*‘Who takes responsibility for the kitchen? For filing the cupboards with clothes for the residents? And then the utility room, which is separate, with garbage and laundry… clean the utility room, there are many clothes that needs to be washed, folded and put away in the rooms… who take care of that?’*

315 *Theme 4: Scope of Practice in Relation to the Residents’ Fundamentals of Care*
316 The fourth theme, ‘Scope of Practice in Relation to the Residents’ Fundamentals of Care (FoC)’, reflected a sense
317 of mutuality between RNs and NRNs, yet also revealed substantial variation and some concerns within the single
318 characteristic that constituted this theme: The What's.

319

320 Both RNs and NRNs described their activities and tasks—their what’s, or functional roles—in ways that
321 predominantly focused on residents’ physical needs. For the RNs, their scope of practice in relation to FoC was
322 described as having a strong somatic focus, with medication management consistently prioritized.
323 In contrast, the NRNs’ descriptions varied more substantially and demonstrated a broader engagement with the
324 psychosocial and relational dimensions of care. While their activities in the physical domain included assisting with
325 hygiene, dressing, nutrition, and monitoring medication, NRNs also emphasized contributions within the
326 psychosocial and relational domains. These included ensuring residents’ well-being, offering emotional support—
327 such as holding hands, showing compassion, providing comfort and building trust—and involving relatives in the
328 care process (Box 8).

329

Dyad 5

RN	NRN

330

331

*‘It is really about dealing primarily with the fundamentals, then. Not so much with how you address other needs outside of the physical, right… So, it's just about the fundamental needs. Elimination, sleep, food, nutrition... Not so much about the... The other type… In a way... I'm not saying that the other needs are not important…*’

Box 8. The what’s *(of scope of practice related to FoC)*

*‘My role here is to ensure that the residents' needs are met... Mostly, or all of their needs. That includes personal care, feeding, activities, the social aspect, communication, talking... And with relatives, when they come…’*

332 RNs and NRNs shared a mutual concern regarding the what’s—the activities and tasks—that reflected a significant
333 shift in the nursing home population, with residents now being older and frailer than in previous years. This shift
334 was particularly notable when considered alongside their earlier accounts in the theme ‘The Context of Practice’,
335 where they described challenges such as inadequate staffing and a lack of appropriate skill mix. These systemic
336 limitations were seen as increasingly problematic in light of the growing number of residents requiring care across
337 all dimensions of the Fundamentals of Care (FoC)—namely, physical, psychosocial, and relational needs.

338

339 Among all identified themes, ‘Scope of Practice in Relation to the Residents’ Fundamentals of Care’ revealed the
340 largest incongruency between RNs’ and NRNs’ what’s, i.e., the specific activities and tasks they reported engaging
341 in. (See pattern chart inspired by Waigwa et al., 2018 [35] – Table 3 – based on the Fundamentals of Care
342 framework [8, 9]

343

344 Table 3.	Pattern chart of interview codes for scope of practice in relation to the fundamentals of care framework [8, 9]

Fundamentals of Care dimensions

Dyad I	Dyad II	Dyad III	Dyad IV	Dyad V	Dyad VI	Dyad VII	Dyad VIII

*RN	NRN	RN	NRN	RN	NRN	RN	NRN	RN	NRN	RN	NRN	RN	NRN	RN	NRN*

*FoC activities related to psychical needs:*

| - Rest and sleep - Personal cleansing and dressing |  | ✘ | ✘ | ✘  ✘ |  | ✘  ✘ |  | ✘ |  |  |  |  |  | ✘ |  | ✘  ✘ |
| --- | --- | --- | --- | --- | --- | --- | --- | --- | --- | --- | --- | --- | --- | --- | --- | --- |
| - Medication management - Toileting needs - Eating and drinking | ✘ | ✘  ✘ | ✘ | ✘  ✘ | ✘  ✘ | ✘  ✘  ✘ | ✘  ✘ | ✘  ✘  ✘ | ✘ | ✘ | ✘  ✘ | ✘ | ✘ | ✘  ✘  ✘ | ✘ | ✘  ✘ |
| - Comfort including vital parameters, pain breathing and positioning - Safety e.g., risk assessment, risk prevention and hygiene |  | ✘  ✘ |  | ✘ | ✘ | ✘ | ✘ | ✘  ✘ | ✘ |  |  |  |  | ✘  ✘ |  |  |
| - Mobility |  | ✘ |  | ✘ |  |  | ✘ |  |  |  |  |  |  |  |  | ✘ |
| *FoC activities related to psychosocial needs:*   - Verbal and written information adjusted to the patient - Being involved and informed about the care - Respected as a person | ✘ | ✘  ✘ | ✘  ✘ | | ✘ | ✘  ✘ |  | ✘  ✘  ✘ | ✘ | | |  | ✘ | ✘ | ✘ | ✘  ✘ |
| - Get education and information - Values and beliefs being considered and respected - Dignity not being embarrassed or offended - Emotional wellbeing, anxiety, worry or stress - Right to privacy, not speaking above the patient |  | ✘  ✘ | ✘ | | ✘ | ✘ | ✘ | ✘  ✘ | ✘ | | | ✘ | ✘  ✘ | ✘ |  | ✘ |
| *FoC activities related to relational needs:*   - Being emphatic, understanding the patient’s situation - Helping patients to cope, supporting in developing and maintaining coping strategies |  | ✘ |  | |  |  |  |  |  | | ✘ |  | ✘ |  | ✘ |  |
| - Engage with the patient, seeing them as individuals |  |  | ✘ | |  | ✘ |  | ✘ |  | | ✘ | ✘ | ✘ | ✘ | ✘ | ✘ |
| - Supporting and involving family and carers - Working with the patient to set goal | ✘ | ✘ | ✘ | | ✘ | ✘ | ✘ |  | ✘ | |  | ✘ | ✘ | ✘ | ✘ | ✘ |
| - Active listening, showing responsiveness |  |  |  | |  |  |  |  |  | |  |  |  |  |  |  |
| - Helping the patient to stay calm |  |  |  | |  |  |  |  |  | |  |  |  |  |  |  |
| - Being compassionate, show care for the patient - Being physically and mentally present with the patient |  | ✘  ✘ |  | |  | ✘ |  | ✘ |  | | ✘  ✘ | ✘ | ✘ | ✘ |  |  |
| *FoC activities displaying a commitment to care as in a trusting nurse-patient relationship*   - Developing relationship, Inviting, introducing him/herself, friendliness - Focusing, eye contact, body language, interpreting signals - Anticipating patients need before the emerge, reflecting prior experiences - Knowledge about health history, fundamental needs and the patients’ personal | ✘  ✘  ✘ | | ✘ | | | ✘ | ✘  ✘  ✘ | | ✘ | | | ✘ | ✘ | ✘  ✘  ✘ | ✘ | ✘ |
| preferences |  | |  | | |  |  | |  | | |  |  |  |  |  |
| - Evaluating care in dialogue with patient |  | |  | | |  |  | |  | | |  |  |  | ✘ | ✘ |
|  |  | |  | | |  |  | |  | | |  |  |  |  |  |


346

347

348

Abbreviations:

RN = Registered nurse

NRN = non-registered nurse

349

350 The pattern chart helped visualize that RNs predominantly described their scope of practice as aligned with
351 the physical dimension of the FoC framework, with medication management being the most frequently reported
352 activity. In contrast, NRNs described a more varied scope of practice, encompassing activities and tasks across all
353 three dimensions of the FoC framework—physical, psychosocial, and relational. This variation points to a
354 divergence in care priorities and engagement between the two nursing roles within the same care context.

355

356 *Theme 5: Notions About Each Other’s Scope of Practice*
357 The theme ‘Notions About Each Other’s Scope of Practice’ reflected varied and incongruent descriptions, as well
358 as concerns regarding how RNs and NRNs experienced and described each other’s scope of practice. Three
359 overarching characteristics constituted the theme: The ‘Other’s’ Whys, The ‘Other’s’ What’s, and Absence vs.
360 Presence.

361

362 The RNs’ descriptions of the other’s whys—that is, the NRNs’ responsibilities in relation to their own—reflected
363 concerns related to their own vague and unclear role. The RNs described a struggle to articulate their role, using
364 expressions such as: “it is too difficult to explain,” “the role is unclear,” “there’s a lack of boundary,” or “there is
365 not much difference between RNs and NRNs—only medication.” Their experiences also highlighted how limited
366 resources and economic constraints led to task shifting, which further complicated their role. When RNs did
367 describe their responsibilities, it was mainly in terms of having more responsibility overall, such as medication
368 management, maintaining an overview, performing administrative tasks, planning and ordinating care, and
369 conducting more complex or specialised procedures. In contrast, the NRNs’ descriptions of the other’s whys also
370 reflected difficulty in articulating the RNs’ role, with some suggesting the RNs depended on the NRNs in practice,
371 while others expressed that NRNs themselves relied on RNs’ clinical knowledge. There were also descriptions
372 indicating that NRNs sometimes had to take responsibility for activities outside of their expected role, even though
373 the RNs were still involved in point-of-care activities (Box 9).

Dyad 7

RN	NRN

*‘I feel that in my role, it’s not just about the residents, but I also have a role with my colleagues, in terms of guiding them and being able to train them in these things. I think that knowledge is power, and it should be shared… When I am in the ward, I have the same function as them…* [*i.e. NRNs*] *’*

*‘Nurses have more knowledge than healthcare workers. That’s what I think. I believe that maybe there is not a very big difference, perhaps just a small difference between nurses and healthcare workers, because even nurses here also have roles related to residents’ nutrition and activities for residents, as we collaborate with them, right?’*

374 Box 9. The *‘*other´s*’* why´s

375

376 Descriptions of the other’s what’s—that is, the activities and tasks performed—also reflected inconsistencies. The
377 RNs predominantly focused on their own scope of practice, especially the preparatory work surrounding physician
378 visits. They described activities such as preparing cases, managing medication, taking bloodwork, informing about
379 residents’ status, and executing delegated tasks. Some RNs expressed frustration over being assigned to what they
380 perceived as less relevant tasks, and a concern that NRNs assumed they were only “sitting in front of the
381 computer” rather than actively preparing for physician visits or revising care plans.

382

383 The NRNs’ descriptions of the RNs’ activities mostly aligned with those of the RNs, including involvement in
384 medication management and physician-related preparations. NRNs acknowledged that RNs had a greater
385 workload but also noted that RNs were less visible in daily care. When NRNs described their own activities in
386 relation to the RNs, they emphasised monitoring, documenting, and relaying information to the RNs, as they were
387 more consistently present with the residents (Box 10).

388

Dyad 8

RN	NRN

389

390

*‘Most is done by healthcare workers, so they inform me so that I can come and administer medication if necessary. Yes, but they are the ones who mostly talk to and calm the residents, and ensure that the psychosocial situation is managed… healthcare workers are the ones who mostly talk to the residents and build that relationship with them…’*

Box 10. The *‘*other´s*’* what´s

*‘Nursing practice, yes... We also have very good cooperation with them* [*i.e. RNs*]*, and it is about helping each other, of course. I think it's fine when we call, for example, if a resident is unwell, if a resident needs some medication or extra painkillers like strong medications. We can't give the strong medications, they come, or they bring the medications. That's what we have. Good cooperation’*

391 The final characteristic of this theme, Absence vs. Presence, reflected experiences of physical presence in the ward
392 and closeness to care. RNs described frequently working alone, being less physically present on the units, and
393 having responsibility for many residents—sometimes for an entire unit, or even the whole nursing home during
394 weekends. This limited presence made it difficult for them to engage directly in care. The NRNs shared similar
395 experiences, describing the RNs as less present during shifts, and more distanced from daily resident interaction.
396 NRNs described that RNs might come into the unit briefly, only when called, and otherwise remained largely
397 absent. This dynamic reinforced the perception that NRNs were the ones consistently closest to the residents (Box
398 11).

399

Dyad 1

RN	NRN

400

401

402

*‘I might not be the one who are the most present in the department and in the care, that you are not present in all the care situations that some might feel you should be’*

Box 11. Absence vs. presence

*Theme 6: Challenges Related to the ‘Others’*

*‘The RNs are with the residents too, but I feel that it is we healthcare workers who are more with the resident throughout the day. It is we who observe more. We are more with the residents then... The RNs come, maybe only if we need help. Or if there is something, then we call, and they might come for a minute or two and then they leave again.’*

403	The final theme, ‘Challenges Related to the Others’, was identified as a unique theme, emerging solely from the

404	RNs' experiences. Although this theme was not reflected in the NRNs’ accounts, it was considered too significant

405	to omit, given its implications for collaborative practice and care quality. The theme centred around one key

406	characteristic: not stronger than our weakest link.

407

408 This characteristic described the RNs’ experiences of dependence on NRNs when accountability was transferred,
409 such as when delegating clinical activities or care responsibilities. RNs described being acutely aware of the varying
410 skills and competencies among NRNs, which could affect their ability to assess situations and make informed
411 decisions. They also emphasised the importance of trust—trusting NRNs to monitor residents accurately, to
412 communicate changes clearly, and to complete delegated tasks properly. The RNs highlighted that, in many cases,
413 they were required to rely on NRNs for key aspects of resident care—particularly given persistent RN shortages—
414 and that this created a vulnerability in the system. One RN expressed this dependence, and the challenges
415 associated with trust in the following way:

416

417	*“A healthcare worker who has taken a course so that they are allowed to administer medication, you can’t go and*

418	*check them afterwards, you have to trust that it is done.” (Dyad 2, RN)*

419

420 The theme also encompassed RNs’ descriptions of how delegation often occurred as a response to understaffing.
421 Activities described as “minor”—such as wound care, basic clinical observations, medication management, and
422 addressing psychosocial and relational needs—were commonly delegated. However, some RNs found delegation
423 difficult, noting that certain NRNs resisted accepting tasks that they perceived as outside their role. These
424 challenges were particularly evident in relation to the organisational context, and sometimes caused frustration
425 for the RNs:

426

427	*“That's the problem because... It is also a challenge in terms of management. Because there aren't many healthcare*

428	*workers who accept tasks from us... They say that it is not their job. Many say that. ‘Those are nursing tasks. It is*

429	*not so positive, when we delegate tasks to them, it is challenging…” (Dyad 5, RN)*

430

431	This theme underscored a critical concern among RNs: while they were ultimately responsible for resident care,

432	their ability to fulfil this responsibility was dependent on the skills, cooperation, and willingness of the NRNs to

433	whom tasks were delegated. In this way, the RNs expressed a vulnerability in their scope of practice—highlighting

434	that they were, in effect, “not stronger than the weakest link.”

435

436 Discussion
437 Using the framework method, we identified six overarching themes: (1) the context of practice, (2) the nurses’
438 professional stance, (3) the scope of practice in relation to residents’ general care, (4) the scope of practice in
439 relation to residents’ fundamentals of care, (5) notions about the other’s scope of practice, and (6) challenges
440 related to the other. These themes reflected the nurses’ (when the term nurses are used here, it includes both
441 RNs and non-RNs) experiences of their individual and mutual general scope of practice, as well as their scope of
442 practice related specifically to the fundamentals of care for older adults in nursing homes. Each theme was
443 composed of multiple sub-characteristics that offered nuanced insights into the nurses' experiences. Below, we
444 focus on the most salient finding.

445

446 Unsurprisingly, the professional practice environment emerged as a key factor in both constraining and shaping
447 i.e., modulating the nurses’ scope of practice, including their responsibilities and activities. A range of
448 environmental modulators characterized the nurses’ experiences, such as excessive administrative tasks,
449 repetitive routines, inadequate staffing levels, an imbalanced skill mix, and overall workplace stress and
450 frustration. While these findings align with existing literature, it is noteworthy—and concerning—that despite
451 decades of research highlighting the influence of such factors, they remain persistent and detrimental to nursing
452 practice. Work values, job satisfaction, and a supportive environment conducive to high-quality care are critical
453 components of an effective professional practice environment [36]. Pursio et al. [37] further argue that supportive
454 environments are built upon elements such as strong leadership, adequate staffing and resources, collegial
455 relationships, and clearly defined quality standards embedded within organizational structures. Nevertheless,
456 recent studies [12, 38, 39] continue to demonstrate that resource constraints—including limited time, inadequate
457 staffing, and poor skill mix—negatively influence nurses’ scope of practice, to the extent that meeting quality
458 standards may become unfeasible within a “context of scarcity” (p. 10) [38]. Conceptualizing nursing as a “systems
459 event” [40] highlights that scope of practice is not a static set of tasks, but rather a dynamic outcome of
460 interactions within a complex care system. It is therefore essential to further explore and address the systemic
461 and environmental modulators that increasingly appear to undermine the scope and quality of nursing practice—
462 particularly those detrimental to patient safety and care standards.

463

464 Our findings clearly indicate that nurses’ professional stance is intrinsically shaped—and constrained—by the
465 quality of the surrounding practice environment. The RNs frequently described being compelled to simplify their
466 responsibilities and activities, including elements of their scope of practice, even when they were aware of the
467 expectations associated with their roles. This dissonance often led to feelings of guilt and inadequacy. Such
468 experiences resonate with the concept of moral distress, which occurs when individuals are unable to act in
469 accordance with what they believe is right due to contextual barriers, thereby compromising their moral integrity
470 [41, 42]. In recent years, the practice environment has increasingly been identified as a key modulator of missed
471 nursing care [43]. Tan et al. [44] further suggest that nurses experiencing moral distress are likely situated in
472 environments that negatively influence their ability to practice professionally. The narratives surrounding nurses’
473 professional stance in our study suggest a potentially adverse effect on their professional identity, here
474 understood as the foundational structure of the nursing profession—including ethics and values, knowledge,
475 leadership abilities, and professional comportment [45]. Supporting nurses in reinforcing their self-esteem may
476 be a critical strategy to prevent burnout and psychological distress—phenomena that remain prevalent among
477 nurses globally [46]. Therefore, strengthening professional identity could serve as a vital entry point to restoring
478 nurses' confidence in their scope of practice. This may be particularly important in situations where there is
479 a mismatch between role expectations or a sense of professional isolation—both of which have been shown to
480 negatively impact the development and maintenance of a strong professional identity [47].

481

482 Consistent with the existing body of literature, our findings reaffirm the well-established distinction between RNs
483 and non-RNs in terms of their engagement with direct and indirect care activities [48], as shaped by their
484 respective scopes of practice in relation to residents’ general care. RNs frequently described their limited presence
485 in the units and in direct care due to their responsibilities in managing and coordinating care, conducting clinical
486 assessments, and overseeing medical and administrative tasks. Their absence from bedside care was largely
487 attributed to time constraints, which they found frustrating. In contrast, non-RNs reported being constantly
488 present and engaged in direct resident care. This contrast can be explained through the ongoing shift that places
489 RNs “further away from the bedside,” often performing indirect care tasks such as medication management, care
490 planning, and documentation. Previous research has noted that RNs often spend a substantial portion of their
491 time on non-value-added tasks—such as searching for supplies, restocking consumables, or locating colleagues—
492 rather than using their expertise in direct care provision [49]. This results in suboptimal utilization of RNs’ full scope
493 of practice [50], while simultaneously increasing their administrative and medical responsibilities [51]. These
494 findings underscore the need for clearer delineation and streamlining of the RN scope of practice to prioritise
495 clinical, supervisory, and care-coordinating functions, while reducing non-essential, non-clinical workload.

496

497 A notable divergence in care priorities also emerged in relation to the fundamentals of care. RNs reported that
498 their scope of practice in this domain predominantly focused on somatic and technical activities, such as
499 treatments and delegated medical care, despite their recognition of the importance of holistic care, including
500 psychosocial and relational needs. This apparent prioritisation was not portrayed as a matter of personal choice
501 but rather as a consequence of systemic constraints. In contrast, non-RNs articulated a broader, more person-
502 centered approach to the fundamentals of care, describing their involvement in addressing residents’ physical,
503 psychosocial, and relational needs. Importantly, our findings challenge narratives found in earlier research
504 suggesting that RNs may not view fundamental care as part of their professional remit [9], or that they consider
505 themselves “too clever” or “too posh to wash” [52, 53]. Rather, RNs in this study were “too busy”—overburdened
506 by administrative and indirect care responsibilities—which constrained their ability to provide comprehensive
507 fundamental care. Their awareness of this imbalance and their expressed discomfort with it underscores a
508 conscientious professional stance. The marked divergence in the focus of RNs and non-RNs in the delivery of
509 fundamental care reflects an adaptive response to the increasing complexity and care needs of residents in nursing
510 homes [54]. However, this adaptation appears to be suboptimally managed, leading to further ambiguity in the
511 RN’s role and displacement of their scope of practice in relation to the fundamentals of care[51]. As Kitson et al.
512 [55] argue, it remains essential to reduce non-value tasks, advocate for fundamental care as a core professional
513 responsibility, and develop leadership that champions high-value, person-centred care.

514

515 Our data also highlighted the difficulty nurses had in articulating their roles and responsibilities—particularly those
516 associated with the RN title. Statements such as “it’s too difficult to explain,” “the role is unclear,” or “hard to
517 distinguish RNs from non-RNs” were common. These comments reflect not only external confusion but also an
518 internal uncertainty about professional identity. Non-RNs often acknowledged RNs’ extensive workloads and
519 limited visibility on the unit but lacked a clear understanding of their colleagues’ professional scope. This ambiguity
520 is not unexpected. Role extension, blurred professional boundaries, and increased administrative responsibilities
521 have long contributed to perceptions of vagueness in the RN role [51, 56, 57]. As a result, articulating one’s
522 individual scope of practice—let alone forming a shared understanding with colleagues—becomes increasingly
523 difficult. This suggests an underlying fragility in the internalisation of professional identity among RNs. Given that
524 professional identity is foundational to ethics, knowledge, leadership, comportment, and overall role clarity [45],
525 strengthening this identity may be essential for sustaining effective teamwork, continuity of care, and career
526 longevity [58]. As such, a meaningful research priority would be to explore how nurses working in long-term care
527 settings experience their professional identity, particularly within the context of increasing complexity and
528 systemic strain. A deeper understanding of the enablers and barriers to professional identity development could
529 provide managers and decision-makers with valuable insights to inform interventions aimed at reinforcing identity,
530 optimizing scope of practice, and ultimately improving quality of care in nursing homes.

531

532 Although the delegation of tasks to other healthcare professionals is a fundamental component of the RN's role,
533 our findings indicate that such transfers of accountability are not without challenges. RNs described significant
534 dependency on non-RNs for the execution of care tasks, including those central to their own scope of practice.
535 The theme “challenges related to the other” highlighted the tensions inherent in this relationship, where RNs were
536 required to delegate substantial responsibilities to NRNs, often under less-than-ideal circumstances. Viewing non-
537 RNs as the "operational arm" of RNs [21] underscores how delegation and supervision have become essential
538 components of the RN’s scope of practice [22]. Effective delegation—defined as transferring responsibility to
539 someone capable of executing the task safely and competently—is crucial for maintaining care quality and
540 workflow [22]. Yet, our findings suggest that delegation was frequently complicated by inconsistent task
541 acceptance, concerns over how tasks were executed, and the inherent asymmetry in visibility and availability
542 between RNs and non-RNs. This dynamic has previously been described as a "double-edged sword" [22], where
543 RNs remain accountable for tasks they do not directly perform, contributing to role tension and professional stress.
544 Reluctance to delegate is well documented, particularly where RNs fear negative outcomes yet lack control over
545 task execution. Scholars have therefore called for greater investment in leadership development for RNs, to
546 support safe, effective delegation practices with clear expectations regarding patient outcomes [23, 59]. Our
547 findings support this: enhancing delegation practices could bolster RN–non-RN collaboration and help reposition
548 delegation as a core, constructive element within the RN’s scope of practice—essential for ensuring efficient and
549 high-quality care in nursing homes.

550

551 Methodological Considerations
552 This study followed several methodological strategies to ensure trustworthiness, transferability, dependability,
553 and confirmability [60]. Our recruitment process targeted participants with varied experiences across four distinct
554 nursing home settings, leading to a diverse and contextually rich dataset, as intended in a qualitative descriptive
555 design [27]. The use of focused interviews [28] enabled participants to express their views in a more open and
556 flexible manner compared to structured formats, generating depth and breadth in the data. We analysed the
557 material using the framework method [29, 61], which allowed systematic comparison within and across dyads.
558 Notably, five of the six identified themes reflected distinct yet complementary perspectives from each professional
559 group,	emerging	consistently	across	all	dyads	and	sites.	This	thematic	recurrence	supports
560 the trustworthiness and theoretical sufficiency of our analysis [62]. With a sample size of 16 interviews (8 dyads),
561 our study exceeds the commonly cited threshold at which saturation is likely achieved [63]. Indeed, no new themes
562 emerged after the tenth interview. Still, the transferability of these findings should be considered in light of the
563 specific context: Norwegian nursing homes operating within a Nordic welfare model. Applicability to other
564 countries or care systems should be approached with caution.

565

566 Conclusions
567 To our knowledge, this is the first study to explore the scope of practice from both RNs and non-RNs using a dyadic
568 approach. Previous research typically addresses these roles in isolation. Our findings emphasise the importance
569 of understanding nursing as a practice embedded within a complex system, where different modulators such as
570 context of care deeply influences both individual and shared scopes of practice. The study reinforces the need
571 to clarify and support professional identity in both RNs and non-RNs. The observed dependency of RNs on non-
572 RNs for executing key care tasks—particularly in fundamental care—highlights how systemic pressures and role
573 redefinitions are reshaping the nursing profession in long-term care settings. The RNs’ shift toward technical,
574 medico-delegated, and administrative duties leaves less time for direct engagement with the full spectrum of
575 fundamental care. However, contrary to earlier assumptions, our results suggest this shift is not due to attitudinal
576 resistance but rather systemic overload—RNs are not “too posh to wash” but often simply “too busy” [53].
577 Delegation of care to non-RNs demands more than task transfer—it requires leadership, communication, and
578 mutual understanding of roles and boundaries. Yet, our findings suggest that professional identity, particularly
579 among RNs, remains insufficiently internalised, leading to ambiguity, diminished visibility, and weakened
580 articulation of scope and responsibilities. These factors can impair collaboration, continuity, and care quality. As
581 such, we propose that nursing professional identity should become a focal point of future research and workforce
582 development, particularly in long-term care. Enhancing professional identity may support nurses in articulating
583 their roles, engaging confidently in delegation, and reclaiming a more balanced, value-driven scope of practice.
584 Ultimately, reinforcing identity, role clarity, and delegation skills among nurses is not only beneficial for staff
585 wellbeing but essential for sustainable, high-quality care delivery in nursing homes.

586

587

588 Disclosure
589 This research report is the third strand of the PhD-project FLORENCE [FundamentaLs Of nuRsing carE professional
590 praCticE]. FLORENCE is conducted with multi-methods focusing on nurses’ scope of practice, the fundamentals of
591 care, older people and the context of home- and facility-based care. FLORENCE is performed between 2021 and
592 2026. Prior to submission a GDPR compliant variant of ChatGPT were used to assist with language refinement and
593 sentence structure optimisation in the final draft.

594

595	The authors critically evaluated and revised all content to ensure that the contributions of AI were limited to minor

596	language improvements without affecting the substance of the work.

597

598 Acknowledgements
599 We wish like to express our gratitude to all involved informants and managers at the four nursing homes for their
600 time, openness, and contribution to this study. This study would not emerge without their engagement. This study
601 was shaped by the valuable insights of the professional, public and patient involvement group at “ANONYMISED”
602 at “ANONYMISED” and “ANONYMISED”

603

604 Ethics Statement
605 This study was assessed and given a waiver by relevant authorities in Norway (Regional Ethics Committees ID
606 691021/2024) and the Norwegian Agency for Shared Services in Education and Research (ID 524471/2024) as well
607 as in Sweden (Research Ethics committee, Karlstad University Sweden (ID C2024/55) and the Swedish Ethical
608 Review Authority (ID 2024-000586-01).

609

610 Author Contributions
611 A1: conceptualization, data collection, analysis, writing original draft, writing and editing.
612 A2: methodology, analysis, validation, reviewing, editing and supervision.
613 A3: analysis, validation, reviewing, editing and supervision.
614 A4: analysis, validation, reviewing, editing and supervision.
615 A5: conceptualization, methodology, analysis, reviewing, editing and supervision.

616

617	Conflicts of Interest

618	The authors declare no conflicts of interest.

619

620	Funding

621	Not applicable.

622

623	Data Availability Statement

624	The raw data that supports the findings of this study are not available due to confidentiality and ethical restrictions

625	including data would not be shared. The developed and tested interview guide are available on request from the

626	corresponding author.

627

628 References
629 1.	Malone G, Cliffe C. Framework for remote and isolated practice. CRANAplus, 2018.
630 2.	Lubbe JC, Roets L. Nurses’ Scope of Practice and the Implication for Quality Nursing Care. Journal
631 of Nursing Scholarship. 2014;46(1):58-64. doi: https://doi.org/10.1111/jnu.12058.
632 3.	Jerlock M, Falk K, Severinsson E. Academic nursing education guidelines: Tool for bridging the
633 gap between theory, research and practice. Nursing & Health Sciences. 2003;5(3):219-28. doi:
634 https://doi.org/10.1046/j.1442-2018.2003.00156.x.
635 4.	Abt M, Lequin P, Bobo M-L, Vispo Cid Perrottet T, Pasquier J, Ortoleva Bucher C. The scope of
636 nursing practice in a psychiatric unit: A time and motion study. Journal of Psychiatric and Mental
637 Health Nursing. 2022;29(2):297-306. doi: https://doi.org/10.1111/jpm.12790.
638 5.	Déry J, Paquet M, Boyer L, Dubois S, Lavigne G, Lavoie-Tremblay M. Optimizing nurses' enacted
639 scope of practice to its full potential as an integrated strategy for the continuous improvement
640 of clinical performance: A multicentre descriptive analysis. J Nurs Manag. 2022;30(1):205-13.
641 Epub 20211014. doi: 10.1111/jonm.13473. PubMed PMID: 34532901.
642 6.	Nursing & Midwifery Council. Standards of proficiency for registered nurses. London: Nursing &
643 Midwifery Council,, 2024a.
644 7.	Zwakhalen SMG, Hamers JPH, Metzelthin SF, Ettema R, Heinen M, de Man-Van Ginkel JM, et al.
645 Basic nursing care: The most provided, the least evidence based - A discussion paper. J Clin Nurs.
646 2018;27(11-12):2496-505. Epub 2018/02/06. doi: 10.1111/jocn.14296. PubMed PMID:
647 29399942.
648 8.	Kitson A, Conroy T, Kuluski K, Locock L, Lyons R. Reclaiming and redefining the Fudamentals of
649 Care: Nursing´s respons to meeting patients´ basic human needs. Adelaide: University of
650 Adelaide; 2013.
651 9.	Feo R, Conroy T, Jangland E, Muntlin Athlin Å, Brovall M, Parr J, et al. Towards a standardised
652 definition for fundamental care: A modified Delphi study. J Clin Nurs. 2018;27(11-12):2285-99.
653 Epub 2017/12/27. doi: 10.1111/jocn.14247. PubMed PMID: 29278437.
654 10.	Feringa MM, de Swardt HC, Havenga Y. Registered nurses' knowledge, attitude and practice
655 regarding their scope of practice in Botswana. Health SA. 2020;25:1415. Epub 20201020. doi:
656 10.4102/hsag.v25i0.1415. PubMed PMID: 33240532; PubMed Central PMCID:
657 PMCPMC7669984.
658 11.	Wiggins D, Downie A, Engel RM, Brown BT. Factors that influence scope of practice of the five
659 largest health care professions in Australia: a scoping review. Human Resources for Health.
660 2022;20(1):87. doi: 10.1186/s12960-022-00783-4.
661 12.	Peters M. Time to solve persistent, pernicious and widespread nursing workforce shortages.
662 International Nursing Review. 2023;70(2):247-53. doi: https://doi.org/10.1111/inr.12837.
663 13.	van Schalkwyk MC, Bourek A, Kringos DS, Siciliani L, Barry MM, De Maeseneer J, et al. The best
664 person (or machine) for the job: Rethinking task shifting in healthcare. Health Policy.
665 2020;124(12):1379-86. Epub 20200830. doi: 10.1016/j.healthpol.2020.08.008. PubMed PMID:
666 32900551.
667 14.	Royal College of Nursing. RCN new definition of nursing - background research and rationale.
668 London: 2023.
669 15.	Yang BK, Carter MW, Trinkoff AM, Nelson HW. Nurse Staffing and Skill Mix Patterns in Relation
670 to Resident Care Outcomes in US Nursing Homes. Journal of the American Medical Directors
671 Association. 2021;22(5):1081-7.e1. doi: https://doi.org/10.1016/j.jamda.2020.09.009.
672 16.	Marchetti D, Cosmai S, Xausa P, Galbiati G, Poliani A, Mainardi C, et al. Nursing staffing and skill
673 mix in nursing homes in Italy: An observational cross-sectional study. Geriatric Nursing.
674 2025;62:48-53. doi: https://doi.org/10.1016/j.gerinurse.2025.01.009.
675 17.	Flodgren GM, Meneses J. Unskilled personnel, skill mix, quality of care and patient safety in the
676 healthcare services: a mapping review. Oslo: Norwegian Institute of Public Health, 2017.
677 18.	Savoie C, Rey S, Yokota S, Dallaire C, Kimura S, Takatani S, et al. Fundamental Care's state of
678 knowledge around the world: Where are we now? A scoping review. J Adv Nurs. 2023;79(3):865-
679 84. Epub 20220505. doi: 10.1111/jan.15278. PubMed PMID: 35511714.
680 19.	Muntlin Å, Jangland E, Laugesen B, Voldbjerg SL, Gunningberg L, Greenway K, et al. Bedside
681 nurses' perspective on the Fundamentals of Care framework and its application in clinical
682 practice: A multi-site focus group interview study. Int J Nurs Stud. 2023;145:104526. Epub
683 20230518. doi: 10.1016/j.ijnurstu.2023.104526. PubMed PMID: 37390582.
684 20.	Nursing & Midwifery Council. Standards of proficiency for nusing associates. London: 2024b.
685 21.	Richards DA, Borglin G. ‘Shitty nursing’ – The new normal? International Journal of Nursing

686	Studies. 2019;91:148-52. doi: https://doi.org/10.1016/j.ijnurstu.2018.12.018.

687 22.	Moradi T, Rezaei M, Alavi NM. Delegating care as a double-edged sword for quality of nursing
688 care: a qualitative study. BMC Health Services Research. 2024;24(1):592. doi: 10.1186/s12913-
689 024-11054-4.
690 23.	Wilson NJ, Pracilio A, Morphet J, Kersten M, Buckley T, Trollor JN, et al. A scoping review of
691 registered nurses' delegating care and support to unlicenced care and support workers. J Clin
692 Nurs. 2023;32(17-18):6000-11. Epub 20230506. doi: 10.1111/jocn.16724. PubMed PMID:
693 37149737.
694 24.	Henderson V. Basic principles of nursing care. Washington: American Nurses Publishing; 1997.
695 25.	Griffiths P, Recio-Saucedo A, Dall'Ora C, Briggs J, Maruotti A, Meredith P, et al. The association
696 between nurse staffing and omissions in nursing care: A systematic review. J Adv Nurs.
697 2018;74(7):1474-87. Epub 20180423. doi: 10.1111/jan.13564. PubMed PMID: 29517813;
698 PubMed Central PMCID: PMCPMC6033178.
699 26.	Nordaunet OM, Gjevjon ER, Olsson C, Aagaard H, Borglin G. What about the fundamentals of
700 nursing—its interventions and its continuity among older people in need of home- or facility-
701 based care: a scoping review. BMC Nursing. 2024;23(1):59. doi: 10.1186/s12912-023-01675-1.
702 27.	Sandelowski M. Focus on research methods. Whatever happened to qualitative description?

703	Research in Nursing & Health. 2000;23(4):334-40. Doi: 10.1002/1098-

704	240x(200008)23:4<334::aid-nur9>3.0.co;2-g. PubMed PMID: 107140570.

705 28.	Merton RK. Focused Interview: Free Press; 2008.
706 29.	Collaço N, Wagland R, Alexis O, Gavin A, Glaser A, Watson EK. Using the Framework Method for
707 the Analysis of Qualitative Dyadic Data in Health Research. Qualitative Health Research.
708 2021;31(8):1555-64. doi: 10.1177/10497323211011599. PubMed PMID: 33980102.
709 30.	O'Brien BC, Harris IB, Beckman TJ, Reed DA, Cook DA. Standards for reporting qualitative
710 research: a synthesis of recommendations. Acad Med. 2014;89(9):1245-51. Epub 2014/07/01.
711 doi: 10.1097/acm.0000000000000388. PubMed PMID: 24979285.
712 31.	Nordaunet OM, Gjevjon ER, Aagaard H, Olsson C, Borglin G. Nursing practice in relation to older
713 people’s fundamentals of care in nursing homes: An exploratory design. International Journal of
714 Nursing Studies Advances. 2025;8:100346. doi: https://doi.org/10.1016/j.ijnsa.2025.100346.
715 32.	Emmel N. Sampling and Choosing Cases in Qualitative Research: A Realist Approach.
716 London2013. Available from: [https://methods.sagepub.com/book/sampling-and-choosing-cases-](https://methods.sagepub.com/book/sampling-and-choosing-cases-in-qualitative-)
717 [in-qualitative-](https://methods.sagepub.com/book/sampling-and-choosing-cases-in-qualitative-)research.
718 33.	Amberscript. Machine-made transcription GDPR, ISO 27001, ISO 9001 and TPN secure.
719 Amsterdam2024.
720 34.	Box. Spotlight on data protection California: Box.com; 2021. Available from:
721 http[s://www.box.com/gdpr.](http://www.box.com/gdpr)
722 35.	Waigwa S, Doos L, Bradbury-Jones C, Taylor J. Effectiveness of health education as an
723 intervention designed to prevent female genital mutilation/cutting (FGM/C): a systematic
724 review. Reproductive Health. 2018;15(1):62. doi: 10.1186/s12978-018-0503-x.
725 36.	Ives Erickson J, Duffy ME, Ditomassi M, Jones D. Development and Psychometric Evaluation of
726 the Professional Practice Work Environment Inventory. J Nurs Adm. 2017;47(5):259-65. doi:
727 10.1097/nna.0000000000000476. PubMed PMID: 28422931.
728 37.	Pursio K, Kankkunen P, Mikkonen S, Kvist T. Organizational characteristics of nursing practice
729 environments related to registered nurses’ professional autonomy and job satisfaction in two
730 Finnish Magnet-aspiring hospitals: structural equation modeling study. BMC Nursing.
731 2024;23(1):100. doi: 10.1186/s12912-024-01772-9.
732 38.	Furtado L, Coelho F, Mendonça N, Soares H, Gomes L, Sousa JP, et al. Exploring Professional
733 Practice Environments and Organisational Context Factors Affecting Nurses’ Adoption of
734 Evidence-Based Practice: A Scoping Review. Healthcare. 2024;12(2):245. PubMed PMID:
735 doi:10.3390/healthcare12020245.
736 39.	Mueller CA, Bowers BJ, Kolanowski AM. RNs in nursing homes-It is not always about the

737	numbers. J Am Geriatr Soc. 2024;72(8):2311-8. Epub 20240329. doi: 10.1111/jgs.18898.

738 PubMed PMID: 38553009.
739 40.	Meadows DH. Thinking in systems. Vermont: Chelsea Green Publishing; 2008.
740 41.	Jameton A. Nursing practice, the ethical issues. Englewood Cliffs, N.J: Prentice-Hall; 1984.
741 42.	Hamric AB, Todd BC, and Epstein EG. Development and Testing of an Instrument to Measure
742 Moral Distress in Healthcare Professionals. AJOB Primary Research. 2012;3(2):1-9. doi:
743 10.1080/21507716.2011.652337.
744 43.	Babaei S, Amini K, Ramezani-Badr F. Unveiling missed nursing care: a comprehensive
745 examination of neglected responsibilities and practice environment challenges. BMC Health
746 Services Research. 2024;24(1):977. doi: 10.1186/s12913-024-11386-1.
747 44.	Tan Jr AK, Capezuti E, Samuels WE, Backhaus R, Wagner LM. Intent to stay, moral distress, and
748 nurse practice environment among long-term care nurses: A cross-sectional questionnaire
749 survey study. Journal of Nursing Scholarship. 2024;56(3):430-41. doi:
750 https://doi.org/10.1111/jnu.12953.
751 45.	Landis T, Barbosa-Leiker C, Clark C, Godfrey N. Professional Identity in Nursing Scale 2.0: A
752 national study of nurses' professional identity and psychometric properties. J Prof Nurs.
753 2024;50:61-5. Epub 20231130. doi: 10.1016/j.profnurs.2023.11.004. PubMed PMID: 38369373.
754 46.	Peng Y, Xiao SW, Tu H, Xiong XY, Ma ZJ, Xu WJ, et al. The Impostor Phenomenon Among Nursing
755 Students and Nurses: A Scoping Review. Front Psychol. 2022;13:809031. Epub 20220309. doi:
756 10.3389/fpsyg.2022.809031. PubMed PMID: 35356345; PubMed Central PMCID:
757 PMCPMC8959846.
758 47.	Thompson J, Cook G, Duschinsky R. “I'm not sure I'm a nurse”: A hermeneutic phenomenological
759 study of nursing home nurses' work identity. Journal of Clinical Nursing. 2018;27(5-6):1049-62.
760 doi: https://doi.org/10.1111/jocn.14111.
761 48.	Montayre J, Montayre J. Nursing Work in Long-Term Care: An Integrative Review. Journal of

762	Gerontological Nursing. 2017;43. doi: 10.3928/00989134-20170519-02.

763 49.	McCloskey R, Donovan C, Stewart C, Donovan A. How registered nurses, licensed practical
764 nurses and resident aides spend time in nursing homes: An observational study. International
765 Journal of Nursing Studies. 2015;52(9):1475-83. doi:
766 https://doi.org/10.1016/j.ijnurstu.2015.05.007.
767 50.	Michel O, Garcia Manjon AJ, Pasquier J, Ortoleva Bucher C. How do nurses spend their time? A
768 time and motion analysis of nursing activities in an internal medicine unit. J Adv Nurs.
769 2021;77(11):4459-70. Epub 20210616. doi: 10.1111/jan.14935. PubMed PMID: 34133039;
770 PubMed Central PMCID: PMCPMC8518809.
771 51.	Stewart C, Berta WB, Laporte A, Deber R, Baumann A. Nurses’ work, work psychology, and the
772 evolution & devolution of care provision in nursing homes: A scoping review. International
773 Journal of Nursing Studies Advances. 2023;5:100133. doi:
774 https://doi.org/10.1016/j.ijnsa.2023.100133.
775 52.	Gillett K. A critical discourse analysis of British national newspaper representations of the
776 academic level of nurse education: too clever for our own good? Nurs Inq. 2012;19(4):297-307.
777 Epub 20110817. doi: 10.1111/j.1440-1800.2011.00564.x. PubMed PMID: 23134276.

778	53.	Olesen EM. Are you "too posh to wash"? Nursing. 2004;34(11):57. doi: 10.1097/00152193-

779	200411000-00046. PubMed PMID: 15686316.

780 54.	Huseby BM. Botid i sykehjem og varighet av tjenester til hjemmeboende. Trondheim: The
781 Norwegian Directorate of Health, 2017 Issue No.: 02/2017.
782 55.	Kitson A, Carr D, Feo R, Conroy T, Jeffs L. The ILC Maine statement: Time for the fundamental
783 care [r]evolution. Journal of Advanced Nursing. 2025;81(1):523-36. doi:
784 https://doi.org/10.1111/jan.16108.
785 56.	Harmer V. Are nurses blurring their identity by extending or delegating roles? Br J Nurs.

786	2010;19(5):295-9. doi: 10.12968/bjon.2010.19.5.47062. PubMed PMID: 20335898.

787	57.	Furåker C. Registered Nurses' views on their professional role. J Nurs Manag. 2008;16(8):933-41.

788	doi: 10.1111/j.1365-2834.2008.00872.x. PubMed PMID: 19094105.

789 58.	Goodolf DM, Godfrey N. A think tank in action: Building new knowledge about professional
790 identity in nursing. Journal of Professional Nursing. 2021;37(2):493-9. doi:
791 https://doi.org/10.1016/j.profnurs.2020.10.007.
792 59.	Crevacore C, Jacob E, Coventry LL, Duffield C. Integrative review: Factors impacting effective
793 delegation practices by registered nurses to assistants in nursing. J Adv Nurs. 2023;79(3):885-95.
794 Epub 20220905. doi: 10.1111/jan.15430. PubMed PMID: 36062891.
795 60.	Lincoln YS, Guba EG. Naturalistic inquiry. California: Sage Publications; 1985.
796 61.	Goldsmith LJ. Using Framework Analysis in Applied Qualitative Research. The Qualitative Report.

797	2021;26:2061-76. doi: 10.46743/2160-3715/2021.5011.

798	62.	Varpio L, Ajjawi R, Monrouxe LV, O'Brien BC, Rees CE. Shedding the cobra effect: problematising

799	thematic emergence, triangulation, saturation and member checking. Med Educ. 2017;51(1):40-

800	50. doi: 10.1111/medu.13124. PubMed PMID: 27981658.

801	63.	Guest G, Bunce A, Johnson L. How Many Interviews Are Enough?:An Experiment with Data

802	Saturation and Variability. Field Methods. 2006;18(1):59-82. doi: 10.1177/1525822x05279903.

803

SRQR reporting guideline

[Click here to access/download](https://www2.cloud.editorialmanager.com/pone/download.aspx?id=40557758&guid=7e55eb44-c9b6-441c-ba80-a6694b49301b&scheme=1)

[**Supporting Information** Appendix 1_SRQR.docx](https://www2.cloud.editorialmanager.com/pone/download.aspx?id=40557758&guid=7e55eb44-c9b6-441c-ba80-a6694b49301b&scheme=1)
