# Supplementary material for: Exploring Nurse Dyads’ experiences of scope of practice in nursing homes: A qualitative descriptive study from the FLORENCE project
Source: PLoS One. 2025 Oct 10;20(10):e0334124. doi: 10.1371/journal.pone.0334124 (PMC12513666; doi:10.1371/journal.pone.0334124)
Supplement: Attachment — Submitted filename: PLOS ONE RN NRN Sweden.docx [file pone.0334124.s005.docx]

The publication is qualitative in nature. The survey was conducted among RNs and NRNs. There were eight dyads of registered nurses (RNs) and non-registered nurses (non-RNs) (n = 16) working in four nursing homes in south-east Norway. Data were collected by means of a structured interview, then coded, and a visualisation was created showing the specific areas of intervention implemented by RNs and NRNs.

The abstract contains all the elements required by the APA standard, also keywords reflecting the content of the publication

The introduction shows the importance of the topic in providing care in nursing homes, the importance of skill-mix and introduces the context of differentiating competencies, according to the current Scope of Practice. The purpose is well defined. There are occasional single spaces between hyphens and words that should not be there.

The methodology sufficiently describes the design of the study, the tools used and the collection of data, their coding, also the inclusion and exclusion criteria.

The results are grouped in clear tables and mapping to areas of standardised coding is also shown. Themes identified in the standard are described in relation to the survey results. The text was supplemented with statements from respondents.

In the manuscript, reference is made to bioethics committee approval

The discussion refers to the study variables and to the literature - giving a broader context for understanding the results.

Dyads varied in the case of RN competencies, in one there is an APN, some nurses also had additional courses, which could affect the workload, shallowing competencies, or taking higher competencies from NRNs, it would be worth highlighting in the limitations. The sample was not large, however, it gives a picture of the situation and the direction to follow to ensure patient needs and safety
