# Supplementary material for: Exploring Nurse Dyads’ experiences of scope of practice in nursing homes: A qualitative descriptive study from the FLORENCE project
Source: PLoS One. 2025 Oct 10;20(10):e0334124. doi: 10.1371/journal.pone.0334124 (PMC12513666; doi:10.1371/journal.pone.0334124)
Supplement: Attachment — Submitted filename: Reply reviewers and editor_29.07.25.docx [file pone.0334124.s008.docx]

Dear Academic Editor, Editor-in-Chief, and Esteemed Reviewers,

We would like to begin by sincerely thanking you for your careful, insightful, and timely review of our manuscript, “Exploring Nurse Dyads’ Experiences of Scope of Practice in Nursing Homes: A Qualitative Descriptive Study from the FLORENCE Project” [Manuscript Reference: PONE-D-25-27718].

We have revised the manuscript to the best of our ability in response to the reviewers' valuable feedback. With your guidance, we believe the revised version represents a substantial improvement and is now more suitable for potential publication in PLOS One. In addressing the important and constructive comments from four reviewers, the manuscript has increased in length and now contains approximately 6700 words. All changes made are highlighted in yellow throughout the manuscript for your convenience.

Our revisions are summarised as follows:

**#Responses to Academic Editor Comments:**

**#1.** Style Requirements:

- *We have revised the manuscript in accordance with the PLOS ONE style guidelines and templates.*

**#2.** Restrictions to Data Sharing:

- *Thank you for your comments and suggestions regarding data sharing. We have reviewed the data-sharing requirements and would like to confirm that, due to ethical and legal restrictions, the data underlying this study cannot be made publicly available. The dataset includes potentially identifying and sensitive participant information. Access to the data is governed by the Norwegian Shared Services for Research and Education (SIKT) and the Swedish Ethical Review Authority (Etikprövningsmyndigheten). We have updated the Data Availability Statement in the manuscript accordingly and have included the contact information for both institutions.*

**#3**. Captions for Supporting Information:

- *The file names have been revised to align with the descriptions provided in the “Supporting Information” template.*

**#4.** Reference List:

- *Thank you for raising this important issue. We have cross-checked all references for retractions using the Retraction Watch Database, CrossRef Retraction Search, individual publisher websites, and PubMed (with support from ChatGPT v3.5). To the best of our knowledge, none of the cited references has been retracted. Additionally, we have reviewed the reference list for formatting consistency and corrected any discrepancies.*

**#Reviewer 1:** Ms Anih Kurnia [*Comments from the editor’s mail, no enclosed file identified]*

Dear Ms. Kurnia,

Thank you very much for your encouraging words and valuable comments, which have supported us in revising our manuscript in line with the standards of PLOS ONE. Your feedback is sincerely appreciated.

### **#1. While the study identifies the impact of organizational context and interpersonal dynamics on scope of practice, the discussion could benefit from deeper integration of existing frameworks or models on interprofessional collaboration or team-based care.**

- *Thank you for this thoughtful and important comment. We have carefully considered your suggestion and revised the discussion accordingly. However, we have chosen to retain our current analytical lens and focus, which does not align directly with existing models of interprofessional collaboration or team-based care. While we recognize that incorporating such models could further enrich the discussion, we feel that doing so would shift the emphasis away from our primary research aim. Furthermore, the manuscript has already increased substantially in length. We hope you understand and accept our rationale for maintaining our current focus.*

### **#2. It is recommended to consider limitations regarding potential response bias or power imbalances in nurse dyads (e.g., hierarchical relationships between RNs and ENs), which may influence the openness during interviews.**

- *Thank you for highlighting this crucial point. We agree that power dynamics and hierarchical relationships can significantly influence data collection in dyadic interviews. However, in our study, we conducted individual interviews with each participant to mitigate such biases and ensure open and honest responses. This methodological choice is now clarified more explicitly in the manuscript.*

### ***#3.*** **Although the study mentions theoretical saturation, it would strengthen methodological rigor to describe how saturation was operationalized (e.g., through coding journals or data matrices).**

- *Thank you for this helpful suggestion. We have revised the manuscript as to clarify that saturation was determined when no new codes or themes occurred in our dyadic data matrices. We hope this addition strengthens the methodological transparency of the study.*

### **#4**.The use of AI language tools for editing is transparently disclosed, which is appreciated. Ensure that future use remains compliant with journal policies.

- *Thank you for your support and acknowledgment. We continue to ensure full transparency regarding our use of AI language tools for editing and remain committed to adhering to all journal policies concerning their use.*

**#Reviewer 2:** Dr Ibrahim Ibn Saana *[Comments from enclosed file, no comments in editors mail identified]*

Dear Dr Saana,

We would like to express our sincere gratitude for the time and effort you have dedicated to reviewing our manuscript. Your thoughtful comments have been instrumental in helping us improve the paper and bring it closer to meeting the standards of PLOS ONE.

**#1.** Clarify Role Terminology: Define “non-registered nurse” early in the manuscript, including typical responsibilities and qualifications, to improve accessibility for international audiences.

- *Thank you for this helpful suggestion. We have added clarifying information regarding both non-registered nurses and registered nurses in the Scandinavian context under the section* Participants and Recruitment*. We hope this improves accessibility for a broader international readership*

**#2.** The lack of demographic details—such as age, years of experience, and educational background—hampers deeper interpretation and subgroup analysis.

- *Thank you for pointing this out. The requested demographic information is (and was) presented in* Table 2 *under the* Results *section. Additionally, we have clarified the educational background of each nurse category in the newly revised* Participants and Recruitment *section. We hope this addresses your concern adequately.*

**#3.** Refine Abstract and Conclusion: Simplify complex sentences in the abstract and conclusion, focusing on key messages and main implications to increase clarity and impact.

- *Thank you very much. We have revised the abstract, relevant parts of the methods, discussion, and conclusion sections to improve clarity, readability, and focus on the study’s key messages and implications. We hope these revisions meet your expectations.*

**#4.** Heavy Use of Quotations: While quotations provide depth, the manuscript could improve narrative flow by relocating longer quotes to appendices or paraphrasing where appropriate.

- *Thank you for this thoughtful and balanced comment. While we recognize the concern about narrative flow, we have been highly selective with the quotations used, limiting them to approximately one per thematic characteristic. In qualitative research, such quotations are essential not only for providing depth but also for enabling readers to evaluate the analysis and judge the transferability of findings—especially given our relatively small sample size and the Nordic context. Reducing or relocating these quotations could compromise transparency and the reader’s ability to engage with our interpretation. We hope our rationale for retaining them is acceptable.*

**#5.** Discuss Broader Relevance: Expand the discussion on how the findings may or may not apply to other healthcare systems, especially those outside Scandinavia or with fewer resources.

- *Thank you for this suggestion. We have revised the text under* Methodological Considerations *to reflect this point. As we are mindful of the overall word count—particularly within the discussion—we hope the current level of elaboration will be deemed sufficient.*

**#6.** Create a Dedicated Recommendations Section and articulate strategies for: Enhancing training in delegation and supervision, strengthening professional identity, particularly among RNs and clarifying role boundaries in interdisciplinary care teams.

- *Thank you for this interesting suggestion. While we agree that the issues raised are important, we have opted not to introduce a separate recommendations section. This is in part due to word count constraints and also because such a section is not standard in* PLOS ONE *articles. Instead, we have integrated organisational and clinical implications into the revised* Conclusion *section, and we hope this will be considered sufficient.*

**#Reviewer 3:** Anonymous [*Comments editors mail, no comments for revision in enclosed file identified]*

Dear Reviewer,

thank you very much for taking the time to review our manuscript and for providing insightful comments. Your feedback has been instrumental in improving the quality of our work, and it is greatly appreciated.

**#1.** There are occasional single spaces between hyphens and words that should not be there.

- *Thank you for noting this. We have carefully reviewed the entire manuscript and corrected all instances of incorrect spacing around hyphens.*

**#2.** Dyads varied in the case of RN competencies, in one there is an APN, some nurses also had additional courses, which could affect the workload, shallowing competencies, or taking higher competencies from NRNs, it would be worth highlighting in the limitations.

- *We agree with this observation and have revised the section* Methodological Considerations *to include a discussion of this variation in competencies and its possible implications for the findings.*

**#Reviewer 4:** Assistant Professor Jalal Khan *[Comments from the editor’s mail]*

Dear Assistant Professor Khan,

We would like to take this opportunity to extend our sincere thanks for the time and effort you dedicated to reviewing our manuscript. Your thoughtful and constructive feedback has been invaluable, and we are truly grateful for your insightful and thorough review.

**#1.** Clarifying the "Non-RN" Category: To assist your international readers, you might consider adding a sentence in the Methods section to briefly define the typical qualifications for the "non-registered nurse" roles in the Norwegian context. This would provide valuable context for understanding the team composition.

- *Thank you very much for this valuable suggestion. We have added a clarification under the* Participants and Recruitment *section regarding the typical qualifications and roles of non-registered nurses (non-RNs) in the Norwegian context. We hope this provides the necessary context for international readers and enhances understanding of the team composition.*

**#2.** Highlighting the Dyadic Analysis Rationale: Your use of dyadic analysis is a key strength of the study. To make the rationale even more explicit for readers, you could consider adding a sentence explaining why the dyad was the chosen unit of analysis (e.g., to enable a direct and rich comparison of perceptions from colleagues practicing together).

- *We greatly appreciate your recognition of the value of the dyadic approach. To make the rationale more explicit, we have emphasized the reasoning for selecting the dyad as the unit of analysis in multiple sections of the manuscript: the abstract, the Methods section (under both* Design *and* Data Collection*), the* Analysis *section, and the* Methodological Considerations*. These additions aim to clarify how dyadic interviews allowed for rich, comparative insights between colleagues working closely together.*

**#3.** Reflecting on the Asymmetry of Theme 6: Your finding that Theme 6 ("Challenges Related to the 'Others'") was unique to RNs is fascinating. A point for consideration could be to briefly reflect in your discussion on why this theme might have been absent from the non-RNs' narratives. A sentence or two speculating on their perspective would add another layer to your insightful analysis.

- *We appreciate this thoughtful suggestion. We have now incorporated a brief discussion in the appropriate section of the discussion to reflect on why Theme 6 ('Challenges Related to the "Others"') was absent from the non-RNs' narratives. This addition provides further depth to our analysis by considering potential differences in perspective and structural dynamics between RNs and non-RNs.*

**Below you will find our point-by-point replies to the comments in the enclosed PDF for which we have not been able to identify the actual reviewers number.**

Dear Reviewer,

Thank you very much for your time, thoughtful feedback, and constructive suggestions. Your input has been instrumental in helping us strengthen the manuscript.

**#1. Abstract:**

- Simplify long sentences in the abstract to improve clarity.
- Ensure consistent use of terms like “RNs” and “NRNs.”
- Clarify the meaning of “non-dyadic” in the Methods section.
- Highlight key findings more explicitly in the Results.
- Rephrase vague expressions such as “at the expense of their professional stance.”
- Add one sentence in the conclusion to emphasize practical or policy implications.
- *The abstract has been carefully revised in line with all points raised above. We have simplified sentence structure, clarified terminology (including "non-dyadic"), emphasized key findings more explicitly, and rephrased vague expressions. Additionally, one sentence has been added to the conclusion to highlight practical implications. Thank you for your constructive feedback and support.*

**#2. Ethical statement:** The use of the term "waiver" should be clarified—was the study formally exempted or approved by the ethics committees? Also, the recruitment dates and consent procedure would benefit from minor grammatical refinement (e.g., “between February 28 and June 4, 2024”).

- *We have revised the ethical statements both in the* Methods *section and under the dedicated* Ethical Statement *section before the references. These now clarify the nature of the ethical approvals and include the corrected grammatical format for dates and consent procedures.*

**#3. Introduction:** Some sentences (e.g., lines 73–75) could be simplified for better readability. Also, consider reducing redundancy in lines 85–88 and updating references on task-shifting (lines 91–94). Strengthen the gap statement in lines 107–110 to emphasize the study’s contribution.

- *The introduction has been reviewed and revised accordingly. We simplified dense sentences, reduced redundancy, and reviewed all references. The majority are from 2020–2024 to reflect current literature, with one from 2025 added for relevance. Please let us know if this inclusion is misaligned with editorial expectations, and we will revise it accordingly. The gap statement has also been strengthened to clarify the study’s contribution.*

**#4*.* Methods:** Consider briefly justifying why a qualitative descriptive design was preferred over other qualitative approaches (e.g., phenomenology or grounded theory), especially given the exploratory aim.

- *Thank you for your thoughtful comment. A qualitative descriptive design was selected for its appropriateness in providing a clear, comprehensive summary of participants’ experiences using everyday language. This approach was preferred over other qualitative methodologies such as phenomenology or grounded theory, as the primary aim of the study was exploratory—to obtain an initial understanding of the topic rather than to generate theory (as in grounded theory) or to deeply explore the lived experience (as in phenomenology). Qualitative description offers both flexibility and practical insight while staying close to participants’ accounts, making it well aligned with the study’s objectives. Given the need to accommodate revisions from all four reviewers, we respectfully hope it will be acceptable to retain the current manuscript text without additional modification.*

**#5. Recruitment & participants:** Consider clarifying the term “non-registered nurses,” as these roles may differ internationally. The explanation of participants’ prior involvement in the broader study (lines 128–130) could be streamlined for readability. Also, briefly elaborate on the rationale for dyad selection based on shared work experience (line 133) to strengthen methodological clarity. Correct the grammatical error in line 135 (“studies contextual framing” → “study’s contextual framing”).

- *This section has been revised in accordance with your comments. We have clarified terminology for international readers, streamlined the background explanation regarding participants’ prior involvement in the broader study, elaborated on the rationale for dyad selection, and corrected the grammatical error.*

**#6. Data collection:** The term “non-dyadic” would benefit from brief clarification, as it may be confusing to readers unfamiliar with the concept. The presentation of the interview content and timeline could also be simplified to enhance readability. Additionally, labelling the interview questions as “IQ1” and “IQ2” should be replaced with a narrative format to better align with academic writing conventions.

- *This section has been revised as suggested. We have clarified the term “non-dyadic,” simplified the structure for better readability, and removed labelling such as “IQ1” and “IQ2,” replacing them with narrative descriptions that better align with academic writing conventions.*

**#7. Data analysis:** Consider briefly explaining the rationale for using a dyadic approach. The interrater reliability process is clear but could be simplified. The stage-by-stage breakdown is thorough, though integrating some steps narratively may improve readability. Minor language refinements (e.g., grammar and consistency in terms like “Stage”) are also recommended.

- *We have revised the* Data Analysis *section accordingly. The rationale for using a dyadic approach is now more clearly stated, and the interrater reliability process has been simplified for better readability. We also revised the description of stages to improve flow and consistency.*

**#8. Table 1:** To improve clarity and academic precision, it is recommended to refine the coding labels, standardize the column headings, shorten lengthy transcript quotes, and ensure consistent terminology when referring to non-registered personnel.

- *Table 1 has been revised to the best of our ability. However, we encountered some difficulty interpreting the full intent of this comment. If any part of our revision does not meet expectations, we would be grateful for further clarification and are happy to make additional changes.*

**#9. Discussion I:** The sentences—especially in lines 467–470—are quite dense and lengthy. Minor revision suggestion: Consider breaking up long sentences into two for better readability, especially for international readers. For example: “This dissonance often led to feelings of guilt and inadequacy. Such experiences resonate with the concept of moral distress…”

- *Thank you for this very reasonable suggestion. The paragraph in question has now been revised for clarity and readability, particularly with regard to sentence length and structure.*

**#10. Discussion II:** Lines 497–513 (Priority differences in fundamental care):The discussion already highlights the gap between idealistic expectations and the reality of RN practice. The contrast between “too posh to wash” and “too busy” is particularly effective. However, this section could be strengthened by emphasizing that this issue stems from systemic changes rather than individual attitudes. Suggestion: Add 1–2 reflective sentences to stress that understaffing and task overload affect not only care quality but also the meaning and experience of RN professionalism.

- *This section has been revised to reflect the influence of broader systemic conditions—such as staffing levels and task overload—on RN practice. We have added reflective commentary to underline how these factors impact both the quality of care and professional identity.*

**#11. Discussion III:** Lines 515–530 (Unclear RN role descriptions):_This paragraph is important but somewhat long and includes repetitive points about the ambiguity of the RN role. Revision suggestion: Merge or simplify the sentences around lines 515–518. Consider reinforcing the message with a direct participant quote, for example: “Participants consistently noted confusion regarding role differentiation, with some stating ‘it’s too difficult to explain’ or ‘hard to distinguish RNs from non-RNs.’”

*This section has been revised as suggested. Redundant points have been merged or simplified to improve clarity. As the paragraph already includes two direct participant quotes, we felt that adding additional quotations might disrupt the balance and readability of the text. We hope this approach will be considered acceptable.*

**#12.Conclusions:** The theoretical contribution of this study (e.g., the use of a dyadic method) could be reinforced in the final lines to underline the novelty of the approach. Minor suggestion: Consider adding a sentence such as: “This study contributes to the literature by offering a dyadic perspective on scope of practice, a dimension that has been largely overlooked in prior research.”

- *Thank you for your valuable input. The conclusion has been revised to highlight the theoretical contribution of the dyadic approach. We have incorporated a sentence closely aligned with your suggestion to underscore this novel aspect of the study.*

*Please let us know if further clarification is needed on any point. We are sincerely grateful for the opportunity to revise and improve our manuscript and thank you again for your constructive feedback.*
