# Supplementary material for: Exploring Nurse Dyads’ experiences of scope of practice in nursing homes: A qualitative descriptive study from the FLORENCE project
Source: PLoS One. 2025 Oct 10;20(10):e0334124. doi: 10.1371/journal.pone.0334124 (PMC12513666; doi:10.1371/journal.pone.0334124)
Supplement: Attachment — Submitted filename: Review comments.docx [file pone.0334124.s007.docx]

**Review comments**

**This study focuses on the practice scope experience of nurse portfolio research, examines the paired working relationship between registered nurses and unregistered nurses, and fills the gap in traditional single-role research. Revealing that systemic factors (such as manpower shortage and administrative burden) are the main cause of RN's departure from basic care ("too busy" rather than "too posh to wash"), challenging the existing cognition, has policy significance for optimizing the allocation of nursing resources. A sample size of 16 people (8 pairs of combinations) is reasonable in qualitative research, and the authors also report reaching theoretical saturation. At the same time, the qualitative description design combined with the framework analysis method is applicable to the research objective, and the non-combined interview design (interviewing RN and non-RN separately) effectively avoids bias caused by power differences. The six extracted themes (such as the pressure in the practice environment, role ambiguity, and the transfer of basic nursing responsibilities) are clinically enlightening.**

**However, there are also some issues. For instance, in the participant table, there is a registered nurse with the qualification of "Advanced Practice Nurse". This qualification difference may affect the description of the scope of practice, but the author did not analyze the impact of this factor on the results, which can be regarded as a minor oversight. Looking at the methodology section, the author used the framework analysis method to handle non-combined interview data. This method is suitable for cross-case comparisons. But why choose "non-combined" interviews instead of joint interviews? The author explains that it is to avoid the influence of power dynamics. This reason holds true, but it should be more prominently emphasized in the method section. In terms of writing, the abstract and conclusion have been revised to be more concise. However, the main text is still rather long, with some paragraphs being overly lengthy, such as the section on moral dilemmas in the discussion. PLOS ONE has high requirements for readability and may need to be further streamlined. The most crucial contribution lies in overturning the stereotype that "nurses are reluctant to do basic care", and echoing the systemic factor with the discovery of "too busy rather than too arrogant". This viewpoint is highly valuable for expression, but the discussion section could highlight its policy significance more prominently.**

**The author needs to solve the following problems:**

**Question 1: Compliance with the data sharing policy is a core requirement of PLOS ONE. The author refused to disclose the data on the grounds of "confidential data" and only provided the contact information of the regulatory agency (SIKT/ Swedish Ethics Review Agency).**

**Suggestion: Specific conditions for data access must be provided (such as signing a confidentiality agreement, anonymization processing procedures). Supplement the legal basis for ethical exemption from disclosing data (such as the provisions of the Personal Data Act of Norway).**

**Question 2: Some methodological details need to be clarified. The term "non-dyadic data" is confusing and the distinction from "dyadic interviews" is not clearly defined.**

**Suggestion: Add a sentence in the method section: "Conduct independent interviews with each participant (not in the form of combined interviews) to avoid response embellishments caused by the presence of colleagues and ensure the authenticity of the data."**

**Question 3: There are issues with the fluency of the article's expression. For instance, too many direct quotations (such as Box 1-11) interfere with the fluency of the argument.**

**There are clerical errors in the analysis example of Table 1 (for example, "DUCH" should be "Dyad"); The terms in the entire text lack consistency. (Abbreviation "non-registered nurse"; for the first occurrence, write the full name + parentheses indicating" non-RN ")**

**Question 4: The reason why "Theme 6 (Delegated Challenge) "was only mentioned by RN was not fully explained, and the reasons for the lack of non-RN perspectives (such as differences in power structure) were not analyzed.**

**Suggestion: The discussion paragraph is supplemented as: "Non-RNs may, due to their role positioning (direct executor rather than decision-maker) or structural power differences, perceive less systemic risks related to delegation responsibilities."**
