# Supplementary material for: Exploring Nurse Dyads’ experiences of scope of practice in nursing homes: A qualitative descriptive study from the FLORENCE project
Source: PLoS One. 2025 Oct 10;20(10):e0334124. doi: 10.1371/journal.pone.0334124 (PMC12513666; doi:10.1371/journal.pone.0334124)
Supplement: Attachment — Submitted filename: Reply reviewers and editor_21.08.25R2.docx [file pone.0334124.s010.docx]

**Dear Academic Editor, Editor-in-Chief, and Esteemed Reviewers,**

We would like to once again express our sincere gratitude for the reviewer’s thoughtful engagement and constructive feedback on our manuscript, “Exploring Nurse Dyads’ Experiences of Scope of Practice in Nursing Homes: A Qualitative Descriptive Study from the FLORENCE Project” [Manuscript Reference: PONE-D-25-27718R1]. We have revised the manuscript to the best of our ability in response to the reviewer’s valuable comments. With your guidance, we believe the revised version represents a significant improvement and is now more suitable for potential publication in PLOS ONE.

All changes have been highlighted in yellow throughout the manuscript for your convenience. A summary of the revisions is provided below:

**Response to reviewer #1**

- *Thank you very much for your supportive comments and for having allowed us to better our text – we are very grateful for the time you have invested in our paper.*

**#Responses to reviewer #5:**

**#1.** **Compliance with the data sharing policy is a core requirement of PLOS ONE. The author refused to disclose the data on the grounds of "confidential data" and only provided the contact information of the regulatory agency (SIKT/Swedish Ethics Review Agency). Suggestion: Specific conditions for data access must be provided (such as signing a confidentiality agreement, anonymization processing procedures). Supplement the legal basis for ethical exemption from disclosing data (such as the provisions of the Personal Data Act of Norway).**

- *Thank you for pointing out this important matter, which we acknowledge is complex. We appreciate the opportunity to clarify our approach to data sharing and would like to emphasize that we do not refuse to disclose data. Our intent has always been to balance compliance with PLOS ONE's data-sharing policy and our ethical and legal obligations to protect participant confidentiality. We apologize if our initial submission caused any misunderstanding and have now carefully revisited our data-sharing obligations.*
- *After consulting with the Norwegian Agency for Shared Services in Education and Research (SIKT) and the institutional Data Protection Officer, it has been reaffirmed that participant consent dictates how the data can be shared. In this study, participants were assured confidentiality, anonymity, and that their data would only be accessible to authorized personnel, such as the close-knit research team. Consequently, participants did not provide consent for anonymised data to be uploaded to a public repository (e.g., Open Science Framework). While the eventual shredding of the code key will render the data anonymised, there remains a potential risk of re-identification ("connect the dots"). For this reason, a case-by-case assessment of data access requests must be conducted under the authority of the institutional Data Protection Officer.*
- *To avoid further misunderstandings, we have carefully reviewed qualitative nursing articles previously published in PLOS ONE and found varied practices regarding data sharing for sensitive qualitative data. Based on this, we propose to follow similar practices to ensure compliance with the journal's policies while upholding our ethical obligations^[1]^. Accordingly, we have refined our Data Availability Statement now presented on p. 14/L592 – 600–We sincerely hope that our amendments are up to par for PLOS ONE.*

**#2. However, there are also some issues. For instance, in the participant table, there is a registered nurse with the qualification of "Advanced Practice Nurse". This qualification difference may affect the description of the scope of practice, but the author did not analyse the impact of this factor on the results, which can be regarded as a minor oversight.**

- *Thank you for your valuable comment. You are absolutely correct in noting that education brings different perspectives and descriptions. However, it is important to emphasize that education is not the only factor influencing such descriptions—work experience is also known to have a significant impact. In our study, we aimed to explore and capture descriptions of the phenomenon “scope of practice” from a broad range of perspectives, regardless of years of work experience, gender, or additional education. In other words, we sought to gain insight from “both sides of the coin,” which would not have been possible with a homogeneous sample. This is highlighted under the heading methods as well as in methodological considerations p. 21 L 527 – 529.*

*It is also important to highlight that, in any qualitative analysis, careful consideration must be given to data that do not fit the emerging themes or categories, regardless of the sampling technique used. This is a principle our research team always adheres to during qualitative analysis, and it was no different in this case. Had we encountered data that did not align with the broader themes, we would have returned to the original transcripts to determine whether these responses reflected a unique perspective among certain informants. Any such deviations would then have been transparently addressed in our reporting of the study. In the last revision we added a sentence under the heading methodological considerations (as requested) clarifying this (p. 22/L545-546)*

*However, no such discrepancies were identified in our analysis. The only notable difference we observed was within general theme 6, which included input solely from RNs and was not described by Non-RNs. In retrospect, this appears quite natural. Therefore, we are confident in the overall reasonableness and trustworthiness of our findings.*

**#3. Looking at the methodology section, the author used the framework analysis method to handle non-combined interview data. This method is suitable for cross-case comparisons. But why choose "non-combined" interviews instead of joint interviews? The author explains that it is to avoid the influence of power dynamics. This reason holds true, but it should be more prominently emphasized in the method section.**

- *Thank you for your comment. We have done our utmost to clarify and emphasise the rationale behind our decision to collect non-dyadic data. This has been addressed under the main* Method *section, specifically in the* Data Analysis *subsection (p. 6, lines 136 – 140), as well as in the* Methodological Considerations *section (p. 22, lines 545–546).*
- *Thank you for your suggested lines below but as that was not part of our rationale for choosing the method we hope that you still will evaluate our amendments as sufficient.*

**#4. Some methodological details need to be clarified. The term "non-dyadic data" is confusing and the distinction from "dyadic interviews" is not clearly defined. Suggestion: Add a sentence in the method section: "Conduct independent interviews with each participant (not in the form of combined interviews) to avoid response embellishments caused by the presence of colleagues and ensure the authenticity of the data."**

- *Thank you for your comment and suggestion. We have clarified the distinction between “dyadic data” (data collected through paired interviews) and “non-dyadic data” (data collected through individual interviews). This clarification is now included in the* Abstract *(lines 26–27), under the main* Method *section and the* Data Collection *subsection (p. 5, line 122), as well as in the* Methodological Considerations *section (p. 22, lines 530–535). We sincerely hope this has now been made clearer.*
- *We would also like to note that the analytical framework we used applies specifically to the analysis of either dyadic or non-dyadic data and does not itself account for a particular data collection method. Therefore, we have adhered to the terminology supported by the referenced method, and we hope this approach is acceptable.*

**#5.** **However, the main text is still rather long, with some paragraphs being overly lengthy, such as the section on moral dilemmas in the discussion. PLOS ONE has high requirements for readability and may need to be further streamlined.**

- *Thank you. We have carefully streamlined the text throughout, but particularly in the Discussion section, including the paragraph referenced on pages 18–19 (lines 436–445). Combined with the removal of the boxes, these changes have significantly reduced the word count—by appx. 1,700 words. We sincerely hope this revision has improved the overall flow and readability of the manuscript*

**#6.** **The most crucial contribution lies in overturning the stereotype that "nurses are reluctant to do basic care” and echoing the systemic factor with the discovery of "too busy rather than too arrogant". This viewpoint is highly valuable for expression, but the discussion section could highlight its policy significance more prominently.**

- *Thank you for once again highlighting the importance of our findings in relation to prevailing stereotypes. We included some discussion on this point in our previous revision but were cautious not to overemphasise it, given the Nordic context of the study and the sample size. However, we have now added further lines to strengthen the policy relevance of this finding, and we hope the revised section (p. 20, lines 477–486) more clearly reflects its significance.*

**#7.** **There are issues with the fluency of the article's expression. For instance, too many direct quotations (such as Box 1-11) interfere with the fluency of the argument.**

- *Thank you for the comment. We are very sorry to hear that our effort to support the results section with relevant quotes may be perceived as interfering. In our previous revision, we acknowledged this concern and explained our intention behind including the quote boxes—namely, to provide a clear rationale and enhance transparency. As this concern was raised by only one reviewer, we also sought editorial guidance on how best to proceed.*
- *For this revision, we have removed all quote boxes from the main text and compiled them into a supplementary information (S2 Table). While we have done this with some reluctance, as we believe the quotes add meaningful context to the findings, we sincerely hope this solution is acceptable.*

**#8.** **There are clerical errors in the analysis example of Table 1 (for example, "DUCH" should be "Dyad"); The terms in the entire text lack consistency. (Abbreviation "non-registered nurse"; for the first occurrence, write the full name + parentheses indicating" non-RN ")**

- *All text, tables, and boxes have been carefully reviewed and revised in accordance with the comments regarding terminology. The abbreviations RN, Non-RN, SoP, and FoC are now consistently used as per the suggestions. These abbreviations are introduced under the relevant headings in the* Introduction*—specifically, under* For Nurses*,* Scope of Practice*, and* Fundamentals of Care*. In line with standard practice, these terms are written out in full in the* Abstract *and* Aim *sections.*
- *The use of the term* dyad *has been reviewed and standardized throughout the manuscript, including in the main text, tables, and boxes.*
- *We conducted a thorough search of the manuscript, tables, and boxes for the term “Duch” but were unfortunately unable to locate its occurrence. We would be grateful for further clarification regarding its location or context so that we can address this appropriately.*

**#9.** **The reason why "Theme 6 (Delegated Challenge) "was only mentioned by RN was not fully explained, and the reasons for the lack of non-RN perspectives (such as differences in power structure) were not analysed. Suggestion: The discussion paragraph is supplemented as: "Non-RNs may, due to their role positioning (direct executor rather than decision-maker) or structural power differences, perceive less systemic risks related to delegation responsibilities."**

- *Thank you for your valuable comment. We have incorporated your excellent suggestion into the relevant part of the Discussion, which we had aimed to address previously. The revised section can be found on p. 21, lines 502–519.*

1. Atakro CA, Atakro A, Aboagye JS, Blay AA, Addo SB, Agyare DF, et al. (2021) Older people’s challenges and expectations of healthcare in Ghana: A qualitative study. PLoS ONE 16(1): e0245451. https://doi.org/10.1371/journal.pone.0245451
