# Supplementary material for: Exploring Nurse Dyads’ experiences of scope of practice in nursing homes: A qualitative descriptive study from the FLORENCE project
Source: PLoS One. 2025 Oct 10;20(10):e0334124. doi: 10.1371/journal.pone.0334124 (PMC12513666; doi:10.1371/journal.pone.0334124)
Supplement: Attachment — Submitted filename: Review comments2.docx [file pone.0334124.s009.docx]

**Review comments**

This study adopted a qualitative descriptive design and employed non-dyadic interviews and frame analysis methods. The differences in experience between registered nurses (RNs) and non-registered nurses (non-RNs) in terms of Scope of Practice (SoP) in nursing homes and their influences on Fundamentals of Care (FoC) were explored. The research design is reasonable, the methods are transparent, the data analysis is rigorous, and the results have clinical and policy significance. The author responded carefully to and revised the comments from the previous round of review, and the quality of the paper has significantly improved. However, there are still some problems. It is suggested that they be further revised：

1.The current discussion section seems to be repeating the results rather than explaining their profound meanings, with the aim of transforming descriptive content into analytical arguments.

2. It is suggested that the discussion section be further streamlined, especially longer paragraphs, to avoid repetitive expression and enhance readability.

For example, the original text example (P. 23, Line 403-406): "In this way, the RNs expressed a vulnerability in their SoP highlighting that they were, in effect, 'not stronger than the weakest link.' Thus, RNs expressed a vulnerability in their SoP, Youdaoplaceholder0 they were "not stronger than the weakest link." (remove redundant phrases such as in this way and in effect)

3. Add a sentence clearly stating in the Method why unpaired interviews were chosen (to avoid the influence of colleagues' presence) to enhance the transparency of the method.

4. Correct minor grammar and word usage errors.

For example, the original example (P. 49, Line 246): The theme "Context of practice" also highlighted environmental challenges faced the nurses. "The theme “Context of practice” also highlighted environmental challenges **faced by** the nurses."

Original (P. 44, Line 105): Recruitment and Informatics

Modification suggestion: Change the title to **Recruitment and Participants** or **Recruitment and Informants**. "Informatics" usually refers to "information science", and using it here is obviously a word mistake.
