# Supplementary material for: Exploring Nurse Dyads’ experiences of scope of practice in nursing homes: A qualitative descriptive study from the FLORENCE project
Source: PLoS One. 2025 Oct 10;20(10):e0334124. doi: 10.1371/journal.pone.0334124 (PMC12513666; doi:10.1371/journal.pone.0334124)
Supplement: Attachment — Submitted filename: Reply_Reviewers_PLOS ONE_R3_V2.docx [file pone.0334124.s011.docx]

**Dear Academic Editor, Dr. Ahmed Abdelwahab Ibrahim El-Sayed, and Esteemed Reviewers,**

We would like to once again express our sincere gratitude for your thoughtful engagement and constructive feedback on our manuscript, “Exploring Nurse Dyads’ Experiences of Scope of Practice in Nursing Homes: A Qualitative Descriptive Study from the FLORENCE Project” [Manuscript Reference: PONE-D-25-27718R3].

In response to the reviewers’ comments, we have thoroughly revised the manuscript with a particular focus on enhancing clarity, language, and grammar. We have addressed all points raised to the best of our ability, and we hope the revised version now meets the expectations for publication in PLOS ONE.

We respectfully request the academic editor, Dr. Ahmed Abdelwahab Ibrahim El-Sayed, to kindly advise on the relevance and implications of Reviewer #7’s comment, particularly in light of the revisions and clarifications we have now provided.

All changes made in this round of revision have been clearly highlighted in yellow throughout the manuscript for your convenience. A summary of these revisions is provided below.

Thank you once again for your continued guidance and support.

**RESPONSE TO THE REVIEWERS**

**Reviewer #5:** **states No on this question while the other reviewer’s states yes**.

*Have the authors made all data underlying the findings in their manuscript fully available? The* [*PLOS Data policy*](http://track.editorialmanager.com/CL0/http:%2F%2Fwww.plosone.org%2Fstatic%2Fpolicies.action%23sharing/1/010f01995bf9ec63-902c9217-b8f8-42aa-82cb-9d72b8aaaccc-000000/ZnESu5FoBF54qb2gnEX8OXXEmDZoQVHdnDFrf7RDPoY=229) *requires authors to make all data underlying the findings described in their manuscript fully available without restriction, with rare exception (please refer to the Data Availability Statement in the manuscript PDF file). The data should be provided as part of the manuscript or its supporting information or deposited to a public repository. For example, in addition to summary statistics, the data points behind means, medians and variance measures should be available. If there are restrictions on publicly sharing data—e.g. participant privacy or use of data from a third party—those must be specified.*

- *Thank you for pointing out this important matter, which we acknowledge is complex. We appreciate the opportunity to clarify our approach to data sharing and would like to emphasize that we do not refuse to disclose data. Our intent has always been to balance compliance with PLOS ONE's data-sharing policy and our ethical and legal obligations to protect participant confidentiality. We apologize if our initial submission caused any misunderstanding and have now carefully revisited our data-sharing obligations.*
- *After consulting with the Norwegian Agency for Shared Services in Education and Research (SIKT) and the institutional Data Protection Officer, it has been reaffirmed that participant consent dictates how the data can be shared. In this study, participants were assured confidentiality, anonymity, and that their data would only be accessible to authorized personnel, such as the close-knit research team. Consequently, participants did not provide consent for anonymised data to be uploaded to a public repository (e.g., Open Science Framework). While the eventual shredding of the code key will render the data anonymised, there remains a potential risk of re-identification ("connect the dots"). For this reason, a case-by-case assessment of data access requests must be conducted under the authority of the institutional Data Protection Officer.*
- *To avoid further misunderstandings, we have carefully reviewed qualitative nursing articles previously published in PLOS ONE and found varied practices regarding data sharing for sensitive qualitative data. Based on this, we propose to follow similar practices to ensure compliance with the journal's policies while upholding our ethical obligations^[1]^. Accordingly, we have refined our Data Availability Statement now presented on p. 14/L592 – 600–We sincerely hope that our amendments are up to par for PLOS ONE.*

**Reviewer #6:** *The only important point in the article is that in the introduction part there is no mention of the Florence project, which is mentioned in the title.*

- Thank you for your valuable comment. Due to the word limit for the manuscript, we have chosen to include a more detailed description of the project under the “Disclosure” section. We sincerely hope this solution is acceptable. However, if preferred, we are of course happy to reconsider and relocate or shorten other sections of the manuscript to accommodate this information elsewhere*.*

**Reviewer #7*:*** *I cannot seem to understand the idea of the dyad when actually what matters is the perception of the nurses of their roles and a little bit of in relation to the non-RNs. If you want to continue with the concept of the dyad, then the voice of the non-RNs should be equally "heard" in this paper. As it looks now, it is mainly the view of the RNs. I would prefer this as well. If you choose this, you might need to revise the title and everything that pertain to the "dyad"*

- Thank you for sharing your thoughtful reflection on our manuscript. Your comment prompted us to thoroughly re-examine our manuscript, particularly the Results and Discussion sections, in addition to Supplementary Material 2 (SM2), Table 1, and Table 3. Following this comprehensive review, we would like to respectfully clarify the following points:

1. We would like to respectfully clarify that throughout the manuscript, the term nurse is used inclusively to refer to both Registered Nurses (RNs) and non-RN nursing staff (non-RNs), as defined in the Introduction (p. 3, lines 49–50). It is important to highlight that this term appears over 100 times, consistently reflecting that both professional groups are included in the descriptions, results, and discussion. In addition, we explicitly specify “RN” or “non-RN” when distinctions between the two groups are relevant to the analysis
2. **Supplementary Material 2 (SM2):** This document, which provides the quotes underpinning the Results section, clearly demonstrates a qualitative dyadic analysis. Each dyad includes perspectives from both RNs and Non-RNs, with their contributions represented equally.
3. **Table 1:** This table presents an overview of the analytical process and includes quotes from a selection of dyadic pairs. These quotes represent both RNs and Non-RNs, further illustrating the dyadic nature of the analysis.
4. **Table 3:** This pattern chart displays the distribution of interview codes for both professional groups in relation to the Fundamentals of Care Framework. While codes from non-RNs are somewhat more frequent, both groups are distinctly and systematically represented.
5. **Results Section:** In this revised version, we have marked with blue text where we explicitly distinguish between RNs and Non-RNs within each general theme. This visual guide demonstrates that both groups are given equitable space in all general themes—except for the final theme, which emerged exclusively in the RNs’ transcripts. A rationale for including this theme is now provided in the manuscript, explaining its relevance despite the absence of corresponding non-RN descriptions.
6. **Discussion Section:** A similar approach has been applied here. We have used blue text to indicate references to each professional group individually and assert that both RNs and Non-RNs are given accurate and appropriate representation in the analysis.

Having conducted this detailed and reflective revision, we respectfully submit that our analysis maintains a balanced and methodologically sound representation of both professional groups. We are genuinely uncertain as to where the concern regarding methodological imbalance may stem from, given the structure of the data and our analytic approach.

We also wish to note that this is our third substantial revision, during which we have received feedback from no fewer than four reviewers at each round. None have previously raised concerns about the qualitative dyadic analysis. At this point, and with the utmost respect, we would kindly request the academic editor, Dr. Ahmed Abdelwahab Ibrahim El-Sayed, to provide guidance on the relevance and implications of Reviewer #7’s comment in light of the revisions and clarifications we have now provided. We remain fully committed to ensuring the rigor and clarity of our work and are open to making further revisions should the editorial team deem them necessary.

**Reviewer #8:** *This study offers valuable insights into the lived experiences of nurse dyads working in nursing homes, particularly regarding their scope of practice. The qualitative descriptive approach is appropriate for exploring nuanced interpersonal and professional dynamics, and the focus on dyads adds a unique relational lens to the analysis. However, some references appear incomplete. Please revise it and modify (Ref. 1, 6, 8, 15, 18, 21, etc). You can add available at (add website) and accessed at (add date).*

- Thank you very much for your encouraging comment—it is greatly appreciated. As requested, the references have been revised and updated accordingly, and the changes have been highlighted in yellow for your convenience.

**Reviewer #X: A)** *The current discussion section seems to be repeating the results rather than explaining their profound meanings, with the aim of transforming descriptive content into analytical arguments. B) It is suggested that the discussion section be further streamlined, especially longer paragraphs, to avoid repetitive expression and enhance readability. For example, the original text example (P. 23, Line 403-406): "In this way, the RNs expressed a vulnerability in their SoP highlighting that they were, in effect, 'not stronger than the weakest link.' Thus, RNs expressed a vulnerability in their SoP, Youdaoplaceholder0 they were "not stronger than the weakest link." (remove redundant phrases such as in this way and in effect).*

- Thank you for your valuable comment. We have once again done our utmost to revise the discussion section in accordance with your recommendations. The section is now approximately 100 words shorter. All additions and changes are highlighted in yellow; however, please note that removed text is not visible in the tracked changes.
- We have also revised the final paragraph of the results section in line with your suggestion. These changes are likewise highlighted in yellow.

**Reviewer #X:** *Add a sentence clearly stating in the Method why unpaired interviews were chosen (to avoid the influence of colleagues' presence) to enhance the transparency of the method.*

- Thank you. The text on page 5, lines 124–125 has been revised and highlighted in yellow.

**Reviewer #X:** *Correct minor grammar and word usage errors. For example, the original example (P. 49, Line 246): The theme "Context of practice" also highlighted environmental challenges faced the nurses. "The theme “Context of practice” also highlighted environmental challenges **faced by** the nurses." Original (P. 44, Line 105): Recruitment and Informatics Modification suggestion: Change the title to **Recruitment and Participants** or **Recruitment and Informants**. "Informatics" usually refers to "information science” and using it here is obviously a word mistake.*

- Thank you for your comment. Minor grammatical and wording errors have been corrected, and the entire manuscript has been re-checked for consistency. All changes, including the two examples mentioned above, have been revised and are highlighted in yellow.

1. Atakro CA, Atakro A, Aboagye JS, Blay AA, Addo SB, Agyare DF, et al. (2021) Older people’s challenges and expectations of healthcare in Ghana: A qualitative study. PLoS ONE 16(1): e0245451. https://doi.org/10.1371/journal.pone.0245451
