# Supplementary material for: Exploring Nurse Dyads’ experiences of scope of practice in nursing homes: A qualitative descriptive study from the FLORENCE project
Source: PLoS One. 2025 Oct 10;20(10):e0334124. doi: 10.1371/journal.pone.0334124 (PMC12513666; doi:10.1371/journal.pone.0334124)
Supplement: Attachment — Submitted filename: REVIEWER COMMENTS.docx [file pone.0334124.s004.docx]

REVIEW COMMENTS

Reviewer: Dr. Ibrahim Ibn Saana, Ph.D.

Date: 17/07/2024

**Manuscript Number: PONE-25-D-27718**

**Title: Exploring Nurse Dyads’ Experiences of Scope of Practice in Nursing Homes: A Qualitative Descriptive Study from the FLORENCE Project**

**STRENGTHS**

Innovative Use of Dyads:

The study’s use of dyadic interviews with registered and non-registered nurses offers a fresh methodological perspective. This design enhances understanding of shared and differing experiences of roles within the same care environment.

Robust Analytical Framework:

The application of the Fundamentals of Care (FoC) framework provides a strong foundation for analyzing the physical, relational, and psychosocial aspects of nursing care.

Compliance with SRQR Guidelines:

Adherence to the Standards for Reporting Qualitative Research (SRQR) enhances the study's methodological integrity and transparency.

Depth of Thematic Analysis:

The study presents well-developed themes—such as professional stance, delegation challenges, and role confusion—which effectively capture the complexity of nursing roles in elderly care.

**CONTEXTUAL RELEVANCE:**

By situating the study in the practical realities of Norwegian and Swedish nursing homes, the authors provide valuable insights into how organizational and cultural factors shape care delivery.

Insight into Emotional and Ethical Dimensions:

The study meaningfully connects role ambiguity and underutilization of skills to issues such as moral distress, burnout, and declining professional identity, particularly among registered nurses.

**TIMELINESS**

The research is highly relevant and timely, aligning with global concerns about workforce optimization in aging populations.

It addresses pressing issues such as task-shifting, skill mix, and quality care in long-term care settings—topics that gained urgency during and after the COVID-19 pandemic.

**RELIABILITY**

The methodology is sound, employing qualitative interviews, a dyadic approach, and a well-established analytical framework.

Ethical considerations are well-handled, with participant anonymity and consent clearly addressed.

However, the study's narrow geographic focus (southeast Norway) and small sample size may limit the generalizability of findings to broader healthcare contexts.

**READABILITY**

The manuscript is structured and professionally written, with an academic tone appropriate for a peer-reviewed journal.

Quotations and subheadings enhance the clarity and flow of the text.

Some sections, particularly the abstract and discussion, could benefit from more concise and simplified language for better comprehension, especially among international readers unfamiliar with Scandinavian nursing roles.

**SCOPE**

The scope is clearly defined, concentrating on role boundaries, delegation, and fundamentals of care in nursing home environments.

While the study is focused on the Scandinavian context, the findings are relevant for healthcare educators, policymakers, and managers across various settings.

However, the limited number of participants and the absence of demographic variation may restrict the applicability of findings to more diverse healthcare systems.

**AREAS FOR IMPROVEMENT**

Limited Transferability:

Since the study is grounded in a Nordic welfare model, its relevance may be limited in countries with different healthcare systems, particularly those in low- and middle-income settings.

Unclear Definition of NRN Role:

The term “non-registered nurse” is used throughout the paper without sufficient explanation. A brief clarification early in the text would aid readers unfamiliar with regional role classifications.

Insufficient Participant Information:

The lack of demographic details—such as age, years of experience, and educational background—hampers deeper interpretation and subgroup analysis.

Heavy Use of Quotations:

While quotations provide depth, the manuscript could improve narrative flow by relocating longer quotes to appendices or paraphrasing where appropriate.

Missing Standalone Recommendations Section:

Though the discussion includes important implications, the manuscript would be stronger with a clearly labeled section outlining practical, actionable recommendations.

**RECOMMENDATIONS**

Clarify Role Terminology:

Define “non-registered nurse” early in the manuscript, including typical responsibilities and qualifications, to improve accessibility for international audiences.

Include Participant Demographics:

Present basic information such as job titles, education levels, and years of experience to strengthen context and enrich analysis.

Create a Dedicated Recommendations Section:

Articulate strategies for:

Enhancing training in delegation and supervision

Strengthening professional identity, particularly among RNs

Clarifying role boundaries in interdisciplinary care teams

Discuss Broader Relevance:

Expand the discussion on how the findings may or may not apply to other healthcare systems, especially those outside Scandinavia or with fewer resources.

Refine Abstract and Conclusion:

Simplify complex sentences in the abstract and conclusion, focusing on key messages and main implications to increase clarity and impact.

**CONCLUSION**

This study is a valuable and timely contribution to nursing research, offering unique insights into the scope of practice within long-term care environments. With minor revisions to improve clarity, scope definition, and practical guidance, the manuscript will be well-positioned for publication in a leading journal such as PLOS ONE.
